# The FCGR2A Is Associated with the Presence of Atherosclerotic Plaques in the Carotid Arteries—A Case-Control Study

**DOI:** 10.3390/jcm12206480

**Published:** 2023-10-12

**Authors:** Anna Szpakowicz, Aleksandra Szum-Jakubowska, Anna Lisowska, Marlena Dubatówka, Andrzej Raczkowski, Marcin Czajkowski, Łukasz Szczerbiński, Małgorzata Chlabicz, Adam Krętowski, Karol Adam Kamiński

**Affiliations:** 1 Department of Cardiology, Medical University of Bialystok, M. Skłodowskiej-Curie 24A, 15-276 Białystok, Poland; anna.szpakowicz@umb.edu.pl (A.S.);; 2 Department of Population Medicine and Lifestyle Diseases Prevention, Medical University of Bialystok, Waszyngtona 13a, 15-269 Bialystok, Poland; 3 Department of Informatics, Bialystok University of Technology, Wiejska 45A, 15-351 Bialystok, Poland; 4 Clinical Research Centre, Medical University of Bialystok, M. Skłodowskiej-Curie 24A, 15-276 Białystok, Poland; 5 Department of Endocrinology, Diabetology and Internal Medicine, Medical University of Bialystok, M. Skłodowskiej-Curie 24A, 15-276 Białystok, Poland; 6 Department of Invasive Cardiology, Medical University of Bialystok, M. Skłodowskiej-Curie 24A, 15-276 Białystok, Poland

**Keywords:** atherosclerotic plaques in carotid arteries, carotid atherosclerosis, FCGR2A, rs1801274, cardiometabolic

## Abstract

Background. Atherosclerotic plaques in carotid arteries (APCA) are a prevalent condition with severe potential complications. Studies continuously search for innovative biomarkers for APCA, including those participating in cellular metabolic processes, cell adhesion, immune response, and complement activation. This study aimed to assess the relationship between APCA presence and a broad range of cardiometabolic biomarkers in the general population. Methods. The study group consisted of consecutive participants of the population study Bialystok PLUS. The proximity extension assay (PEA) technique from the Olink Laboratory (Uppsala, Sweden) was used to measure the levels of 92 cardiometabolic biomarkers. Results. The study comprised 693 participants (mean age 48.78 ± 15.27 years, 43.4% males, N = 301). APCA was identified in 46.2% of the participants (N = 320). Of the 92 biomarkers that were investigated, 54 were found to be significantly linked to the diagnosis of APCA. After adjusting for the traditional risk factors for atherosclerosis in multivariate analysis, the only biomarker that remained significantly associated with APCA was FCGR2A. Conclusion. In the general population, the prevalence of APCA is very high. A range of biomarkers are linked with APCA. Nonetheless, the majority of these associations are explained by traditional risk factors for atherosclerosis. The only biomarker that was independently associated with APCA was the FCGR2A.

## 1. Introduction

Carotid atherosclerosis (CA) is defined as the presence of atherosclerotic plaque in the extracranial common carotid artery (CCA), internal carotid artery (ICA), external carotid artery (ECA) or carotid bulb. It is a condition with potentially serious complications, such as stroke or transient ischaemic attack (TIA). Patients with CA are also at increased risk of myocardial infarction, limb ischaemia events or cardiovascular death [1]. The risk of adverse events depends on the degree of carotid artery stenosis (narrowing of the vessel lumen expressed as a percentage). In asymptomatic patients with CA with stenosis ≥ 50%, the predicted risk of ipsilateral stroke is 0.5–1.0% per year [2,3]. In patients with severe CA (stenosis 70–99%) and optimal conservative treatment, the one-year risk of stroke is 0.9% [4]. The SMART (Second Manifestations of ARTerial disease) study included patients with symptomatic arterial disease or type 2 diabetes mellitus but no history of stroke [5]. The 5-year cumulative risk of vascular death, ischaemic stroke or myocardial infarction was 12.3% (stroke 2.2%, myocardial infarction 8%). In multivariate analysis, CA was also independently associated with the risk of cardiovascular death (HR = 1.8) [5].

In the SMART study, asymptomatic CA of ≥50% was found in 8% of subjects. In contrast to patients with diagnosed cardiovascular disease, the prevalence of asymptomatic CA (defined by ≥50% stenosis) is low in the general population. In a meta-analysis involving 23,000 participants, it was observed in 0.2% (95% CI, 0.0% to 0.4%) of men aged <50 years and 0% (0% to 0.2%) of women aged <50 years [6]. However, the percentages were significantly increased in those aged ≥80 years: 5% (3.1% to 7.5%) in women and 7.5% (5.2% to 10.5%) in men [6].

The development of CA is accelerated by a number of factors. Traditional risk factors for carotid atherosclerosis include age, male sex, smoking, diabetes, hypertension, hypercholesterolaemia, obesity, and a sedentary lifestyle. They are analogous to risk factors for atherosclerosis in other vascular beds. However, the prevalence of specific risk factors varies between patients with different sites of atherosclerosis [7]. Patients with carotid atherosclerosis are more likely to have hypertension, diabetes or hypercholesterolaemia [7]. Established risk factors only provide an estimate of the CA probability. Incorporating new risk factors and biomarkers into the model could improve clinical risk assessment. Non-traditional risk factors still under investigation include sleep disturbances, air pollution, stress, or pathological changes in the gut microbiome [8].

The identification of novel candidate molecules for risk factors or risk markers requires knowledge of the mechanisms of atherosclerotic plaque development. It is a long-term complex process that is preceded by endothelial dysfunction (decreased NO availability and increased activity of vasoconstructive agents). It begins with leukocyte adhesion and migration through the vessel wall into the intima and activation of immune processes. Next, smooth muscle cells migrate from the media into the intima and proliferate, and macrophages and smooth muscle cells transform into foam cells. Then, apoptosis of all cells in the atherosclerotic plaque increases, and coagulation is activated [8,9,10]. All these complex processes are conditioned and influenced by multiple factors. Particular attention has been paid to the role of systemic inflammation and inflammatory biomarkers such as hsCRP, IL-6, IL-1, TNF, or IFNγ [8,9,11]. Due to the activation of the pro-inflammatory state, several chronic or acute infections are also associated with atherosclerosis, including periodontal disease, Helicobacter pylori, cytomegalovirus, or pneumonia [12]. 

The cardiometabolic panel developed by the Olink Proteomics Laboratory (Uppsala, Sweden) includes a broad range of biomarkers of cellular metabolism, cell adhesion, immune response, and complement activation that may be potential biomarkers of CA (listed in Supplementary Table S1). The aim of this study was to assess the relationship between the presence of atherosclerotic plaques in the carotid arteries (APCA) and a broad range of cardiometabolic biomarkers in the general population. 

## 2. Material and Methods

The study group consisted of 693 consecutive participants of the Bialystok PLUS (Polish Longitudinal University Study) with a complete database including carotid ultrasound and proteomic cardiometabolic panel. Bialystok PLUS is an ongoing population study that aims to comprehensively assess the health status of registered citizens of the city of Bialystok (including medical history, risk factors for lifestyle diseases, blood tests, heart, vascular, and liver imaging, lung function, or genomics). It comprises participants aged 20 to 80 years old [7]. The exclusion criterion is an acute infection within the previous 6 weeks, based on subjective assessment by the participant. When confirming the examination date (2 days before the scheduled examination), the participant was asked whether he or she had had an acute infection in the previous 6 weeks. If the answer was yes, the examination date was postponed. Participants were randomly selected from the database of the mayor’s office. 

A comprehensive medical history was taken from all the participants, and all subjects underwent a thorough medical examination. After fasting for at least 8 h, blood samples were collected from peripheral veins. Plasma was obtained by centrifugation at 1810 G for 10 min at room temperature. Immediately after centrifugation, biochemical tests were performed in an automated biochemical analyzer (Cobas c111 machine, Roche Diagnostics Ltd., Rotkreuz, Switzerland). Blood count was performed from blood collected in a tube with EDTA anticoagulant (Mythic 18 machine, Orphee, Geneva, Switzerland).

Anthropometric measurements were taken, including height and weight. Body mass index (BMI) was calculated as weight in kilograms divided by height in metres squared. Blood pressure (BP) was measured after the participants had been seated for at least 5 min using an Omron Healthcare Co., Ltd. Terado-cho, Muko, Kyoto, Japan. 

Carotid ultrasound was performed using the Vivid™9 device and a 9L-D linear array probe with a frequency of 10 MHz (frequency range 4–11 MHz, GE Healthcare, Chicago, IL, USA). The study was performed in the supine position by a qualified technician. CCA intima-media thickness (IMT) was measured at the posterior wall of the arteries, approximately 1 cm before the bifurcation, and repeated 5 times on each side. The mean value was then calculated for the left and for the right side. The average of both sides was used for further analysis. The presence of APCA in (1) right CCA, (2) left CCA, (3) right ECA, (4) left ECA, (5) right ICA, (6) left ICA, (7) right bifurcation (BIF), and (8) left BIF was evaluated. APCA was defined according to the Mannheim Intima-Media Thickness and Plaque Consensus as a local structure that encroaches into the arterial lumen at 0.5 mm or 50% of the surrounding IMT or has a thickness >1.5 mm measured from the media-adventitia interface to the intima–lumen interface [13]. 

Diabetes was defined as a history of diabetes or use of antidiabetic drugs or fasting glucose concentration ≥ 7 mmol/L (126 mg/dL) or glucose after 120 min of an oral glucose tolerance test (OGTT) ≥ 11.1 mmol/L (200 mg/dL). Hypercholesterolaemia was defined as a history of hypercholesterolaemia or use of lipid-lowering drugs or a total cholesterol concentration ≥ 4.9 mmol/L (190 mg/dL) or low-density lipoprotein cholesterol (LDL cholesterol) concentration ≥ 3.0 mmol/L (115 mg/dL). Obesity was diagnosed if the BMI was ≥30 kg/m^2^.

For proteomic analysis, plasma samples were stored at a biobank at −80 °C, thawed at 4 °C, prepared and sent to the Olink Proteomics laboratory (Uppsala, Sweden) for analysis. We used a 92-analyte panel—cardiometabolic. This is a proprietary panel developed by Olink that includes a wide range of biomarkers involved in cellular metabolic processes, cell adhesion, immune response, and complement activation (biomarker names listed in Supplementary Table S1). Biomarker levels were measured using the proximity extension assay (PEA) multiplexing method, which is based on quantitative PCR [14]. It does not allow the measurement of the concentration of biomarkers in absolute values, but the relative level of peptides expressed in the NPX unit (Normalised Protein eXpression) is obtained. NPX is specified on a logarithmic scale (binary log, log2). The limit of detection (LOD) was estimated for each sample plate as the concentration in the negative controls plus three standard deviations. Study LOD was defined as the maximum observed LOD among all the plates. For each biomarker, if more than 25% of the samples were below the LOD, the biomarker was excluded from further analysis. For the remaining biomarkers, the values below the LOD were replaced by the respective LOD.

Blood collected in an EDTA tube was used for genetic testing. Preparation was performed under sterile conditions, and samples were stored in a biobank at a temperature of −80 °C. Whole blood samples were sent to the Eurofins Genomics Laboratory (Galten, Denmark) at −20 °C. Genetic testing was performed by the laboratory using the Infinium Global Screening Array v3.0 (Illumina, San Diego, CA, USA). This array allows genome-wide genotyping of 654,027 markers using a high-throughput next-generation sequencing method. The Intelliomics platform was used to prepare and transform the dataset used in the experiments performed [15].

## 3. Ethical Issues

Ethical approval for this study was provided by the Ethics Committee of the Medical University of Bialystok (Poland) on 29 September 2016 (approval number: R-I-002/323/2016). The study was conducted in accordance with the Declaration of Helsinki, and all participants gave written informed consent.

### Statistical Analysis

The Chi-square test was used to compare the categorical variables. The normality of continuous variables was tested using the Shapiro–Wilk test. Data with normal distribution were presented as mean value and standard deviation, and data with other distributions were presented as median with interquartile range ((IQR) 1st quartile–3rd quartile). The homogeneity of variance was tested using the Levene test. Comparisons of continuous variables between subgroups were performed using the T-Student test (variables with normal distribution and homogeneous variance), Mann–Whitney test (other distributions or nonhomogeneous variance), or Kruskall–Wallis test (comparison of 3 subgroups). 

For biomarkers with significant differences in levels between participants with and without APCA, univariate logistic regression was performed with a biomarker as the independent variable and APCA as the dependent variable. If a significant association was found, multivariate logistic regression was performed, and 7 traditional risk factors for atherosclerosis were included in a model as independent variables (age, sex, diabetes, hypercholesterolaemia, obesity, current smoking, and hypertension). A stepwise backward logistic regression model was constructed only if the association between a biomarker and APCA remained significant. For regression analysis with a continuous variable as the dependent one, linear regression was used. 

All calculations were performed using STATISTICA 13.3 software (STATSoft). A *p*-value < 0.05 was considered statistically significant.

## 4. Results

We included in the study 693 participants. They were all Caucasian. The mean age was 48.78 ± 15.27 years; 43.4% were male (N = 301), 93 had diabetes (14.9%), 493 hypercholesterolaemia (71.1%), and 313 had hypertension (45.2%), 193 were obese (27.8%), and 147 were current smokers (21.2%). APCA was diagnosed in 46.2% of the participants (N = 320). APCA with ≥50% stenosis was described in 9 cases (1.3%). Detailed clinical characteristics of the study group based on the presence of are shown in Table 1. Patients with APCA were significantly older, had higher blood pressure and BMI, and were more frequently diagnosed with hypertension, diabetes, or hypercholesterolemia. They also had higher concentrations of fasting glucose, LDL cholesterol, triglycerides, hsCRP, and interleukin-6. Patients with APCA were more frequently treated with all classes of drugs analysed: beta-blockers, ACE inhibitors or sartans, diuretics, hypolipemic agents, antidiabetic agents, and antiplatelet or anticoagulant agents.

The Olink Target 96 Cardiometabolic panel included 92 biomarkers (biomarker names listed in Supplementary Table S1). For 7 biomarkers, more than 25% of the measurements showed values below the limit of detection (marked with * in Supplementary Table S2). These biomarkers were not analysed further. 

We compared the NPX values between the participants with and without APCA. There were significant differences for 56 biomarkers (analysis in columns 3–5, Supplementary Table S2). Next, we validated the association between these 56 biomarkers and APCA diagnosis in univariate logistic regression, with significant results for 54 of them (analysis in columns 6–7, Supplementary Table S2).

**Figure 1:**
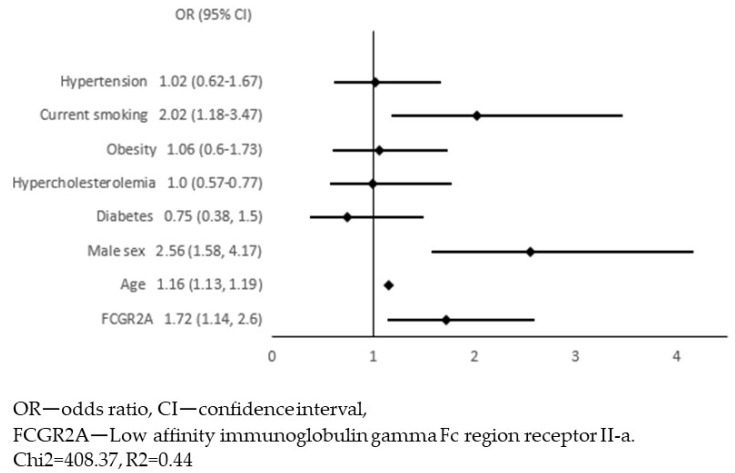
A primary model for multivariate logistic regression with the presence of atherosclerotic plaques in the carotid arteries (APCA) as the dependent variable and FCGR2A level together with traditional risk factors for atherosclerosis as independent variables.

**Figure 2:**
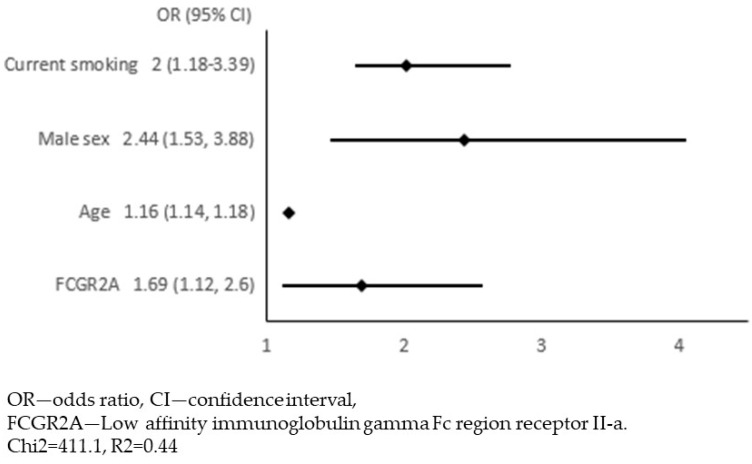
Final model for multivariate logistic regression with the presence of atherosclerotic plaques in the carotid arteries (APCA) as dependent variable and FCGR2A level together with traditional risk factors for atherosclerosis as independent variables.

**Figure 3:**
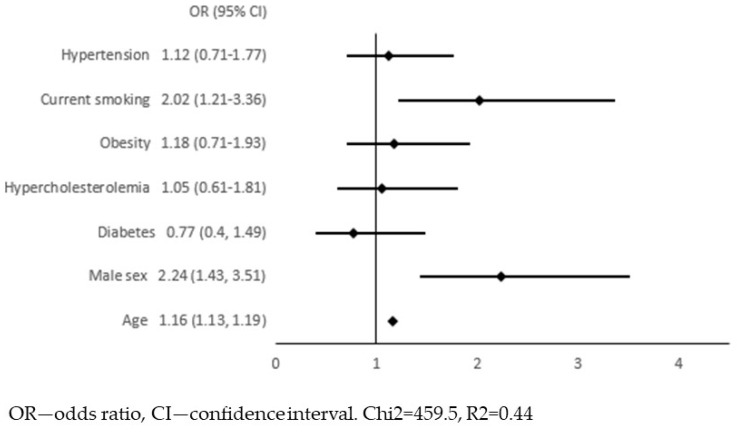
Multivariate logistic regression with the presence of atherosclerotic plaques in the carotid arteries (APCA) as dependent variable and traditional risk factors for atherosclerosis as independent variables.

In the next step, we adjusted these 54 associations for traditional risk factors for atherosclerosis: age, sex, diabetes, hypercholesterolaemia, obesity, current smoking, and hypertension. We constructed 54 models of multivariate logistic regression with the presence of APCA as the dependent variable and one of the biomarkers together with 7 risk factors for atherosclerosis as independent variables (all models presented in Supplementary Material S1). The only biomarker that remained significantly associated with APCA was the FCGR2A (low-affinity immunoglobulin gamma Fc region receptor II-a). In this model, we continued the analysis in order to select variables independently associated with APCA. Variables with a *p*-value ≥ 0.05 were removed from the model in a backward stepwise selection. Figure 1 and Figure 2 show the results of multivariate logistic regression, including FCGR2A and traditional risk factors for atherosclerosis. In the final model (Figure 2), the FCGR2A level was independently and significantly associated with APCA prevalence together with age, male sex, and current smoking. The R2 value for this model was 0.44, and it was similar to the R2 for the model, including all traditional atherosclerosis risk factors without additional biomarkers (Figure 3). 

**Figure 4:**
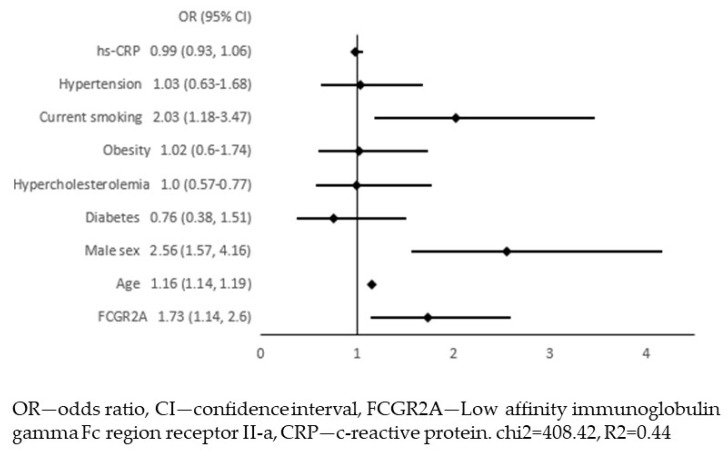
Multivariate logistic regression with the presence of atherosclerotic plaques in the carotid arteries (APCA) as dependent variable and FCGR2A level together with traditional risk factors for atherosclerosis and hsCRP as independent variables.

The FCGR2a acts as a receptor for C-reactive protein (CRP) and is involved in CRP-induced inflammation [16]. Therefore, we included hsCRP in the list of independent variables in the multiple regression. HsCRP did not show a significant association with APCA, nor did it change the final model (Figure 4). The FCGR2A level was also associated with mean IMT as a dependent variable value in linear regression (β = 0.12, *p* = 0.002). 

To gain further insight into the factors influencing FCGR2A, linear regressions were performed with the FCGR2A level as the dependent variable. In the univariate model, the biomarker was associated with age, diabetes, obesity, and hypertension diagnosis (Table 2). In the final multivariate model, the FCGR2A was associated with age and sex (Table 2).

**Figure 5:**
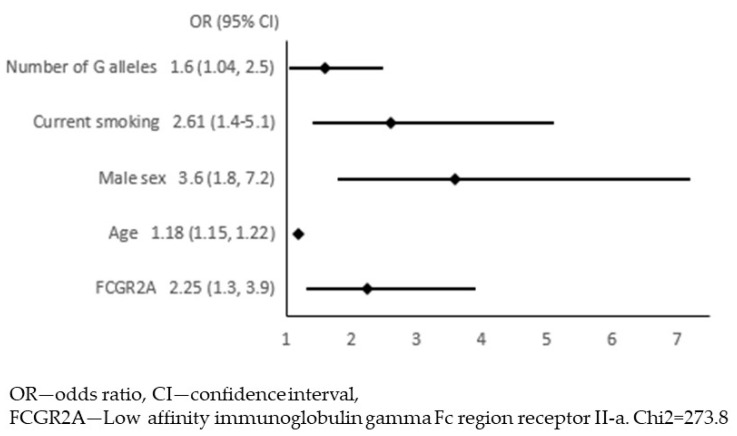
Multivariate logistic regression with the presence of atherosclerotic plaques in the carotid arteries (APCA) as the dependent variable and the FCGR2A level together with traditional risk factors for atherosclerosis and the number of G alleles of the rs1801274 SNP as independent variables.

With GWA assays available, the next step was to analyze the literature for genetic variability of FCGR2A. The most frequently described polymorphism was rs1801274, which is also associated with the occurrence of CA [17]. We analysed it in a group of 675 participants who were successfully genotyped. There were significant differences between the genotypes in the FCGR2A level: median 4.08 (IQR 3.69–4.34) NPX in AA homozygotes, 3.97 (3.63–4.19) NPX in AG heterozygotes and 3.89 (3.66–4.1) NPX in GG homozygotes, *p* = 0.0023 in Kruskal–Wallis test. There was no difference in the percentages of specific genotypes between the patients with and without APCA (Table 3), *p* = 0.37 (chi2 test). However, in multivariate logistic regression, the number of G alleles was one of the variables independently associated with the diagnosis of APCA (Figure 5).

## 5. Discussion

In our study of the general population, a wide range of biomarkers was associated with APCA (54 out of 92 biomarkers), including biomarkers involved in cellular metabolism, cell adhesion, immune response, and complement activation. These results, especially the high number of associations observed, illustrate how complex and multidirectional atherogenesis is and how many molecules are involved in this process. However, it is very significant that all but one of the associations could be attributed to the influence of traditional risk factors for atherosclerosis. This means that the 53 molecules were not markers of APCA itself but markers of, for example, age, diabetes, or obesity. Simple clinical variables formed a model with an R2 value that was not additionally increased by cardiometabolic data. Therefore, our diagnostic and therapeutic targets remain the clinical diagnoses and not the biomarkers associated with them. 

The literature also describes the important role of systemic inflammation and oxidative stress in the pathogenesis of atherosclerosis. In a recent study of patients with type 1 diabetes, hsCRP and myeloperoxidase were useful biomarkers for assessing the risk of carotid atherosclerosis [18]. They could also be considered as potential makers of disease progression despite optimal medical treatment and traditional risk factor control, which in turn is associated with an increased risk of adverse cardiovascular events [19]. 

In our material, the only biomarker that was independently associated with APCA was the FCGR2A. The final multivariate model for predicting APCA in our population included the FCGR2A level, age, male sex and current smoking. The FCGR2A (low-affinity immunoglobulin gamma Fc region receptor II-a, also known as Fc gamma receptor IIa or as CD32) is a cell surface receptor found on macrophages and neutrophils. It binds to the Fc region of immunoglobulins gamma, initiates the cellular response against pathogens and promotes phagocytosis [14,20]. The expression of FCGR2a is increased in several carcinoma tissues [21]. Recent Mendelian randomisation analysis of the blood proteome showed that increased FCGR2A levels decreased the risk of atrial fibrillation, and this receptor could be a novel drug target [22]. This biomarker also plays a role as a receptor for CRP, and in this pathway, it is involved in CRP-induced inflammation [14,16]. In our material, participants with APCA had significantly higher hsCRP levels than those without carotid atherosclerosis. However, when logistic regression was used, this association was not significant, nor did hsCRP alter the final multivariate model (with APCA as the dependent variable and the FCGR2A level together with traditional risk factors for atherosclerosis and hsCRP as independent variables).

The rs1801274 (G>A) is the polymorphism of the FCGR2A gene, known as R131H in the previous literature. Under in vitro conditions, CRP binds more strongly to monocytes and neutrophils derived from RR homozygotes than to monocytes and neutrophils of other genotypes [23]. RR homozygotes are also less effective at clearing IgG2 complexes [23]. In a small case-control study (134 patients with coronary artery disease [CAD] and 251 controls), the R131H single nucleotide polymorphism was associated with the diagnosis of CAD, with the RR being the high-risk genotype [24]. There were 6.5% and 11% of RR homozygotes in controls, compared with 19% in patients with CAD [24]. This genotype remained significantly associated with CAD after adjustment for traditional risk factors in multivariate logistic regression (OR 6.3, 95%CI 1.1, 36.3) [24]. The RR genotype was also more prevalent in patients with acute coronary syndromes compared to patients with stable CAD (OR 2.86, 95% CI 2.06, 3.99) [25]. In the Rotterdam Study, the H allele was protective for advanced peripheral atherosclerosis: OR adjusted for sex 0.65 (95% CI 0.44–0.98) [26]. In the Young Finns Study, the SNP had no effect on IMT [27]. In patients with systemic lupus erythematosus, the R allele carriers had more frequent subclinical carotid atherosclerosis than HH homozygotes (58% vs. 25%, *p* = 0.04). The R allele was also associated with increased platelet activity [28]. All these studies, except the Young Finns Study, were limited by the small number of patients included. In none of them was the FCGR2A level measured. The FCGR2A gene belongs to the glutamate receptor signalling pathway. The glutamate receptor elicits a protective effect in cardiac and cerebral ischaemia by inhibition of the FCGR2A gene [29]. It has been identified as a key protein–protein interaction gene linking the pathogenic pathways of carotid atherosclerosis and periodontitis [17]. The role of the rs1801274 polymorphism in infectious diseases varies depending on the disease. In patients with pneumococcal disease (pneumonia or meningitis), the RR genotype was associated with improved survival [30]. On the other hand, the R variant was more common in septic patients compared with non-septic critically ill patients or healthy controls [31]. In a case-control study, the HH genotype was protective against malaria manifestation [32]. The FCGR2A polymorphism has also been implicated in autoimmune diseases: lupus erythematosus [33], Kawasaki disease [33], rheumatoid arthritis [34] or ulcerative colitis [35].

In our material, there was no difference in the percentages of specific genotypes between the patients with and without APCA; however, in multivariate analysis, the G allele was associated with the diagnosis of APCA. Surprisingly, the GG genotype (considered a high-risk genotype, equivalent to the RR genotype) was associated with lower FCGR2A levels expressed in NPX. On the other hand, patients with APCA had higher FCGR2A levels than those without APCA. It is possible that the adverse effect of the G allele observed in other studies may be independent of the FCGR2A serum concentration. It is important to emphasise that we have measured serum, not cell surface abundance of this protein, and therefore, we cannot draw conclusions about the cellular downstream pathway activity. According to the literature, the adverse clinical effect of the G allele would be due to stronger binding to CRP, increased activation of CRP-induced inflammation or decreased clearance of IgG2 complexes [16,23]. Furthermore, there are no studies that combine the rs1801274 with the FCGR2A plasma level measurement.

In our study, APCA was diagnosed with a very high frequency of 46.2%; however, significant carotid stenosis (≥50%) was found in 1.3% of participants. In comparison, in a large meta-analysis including 59 studies (participants aged 30–79 years), the estimated prevalence of APCA was 21.1%, and the estimated frequency of significant carotid stenosis was 1.5% [36]. In a Chinese study (comprising >100,000 participants, mean age of 60.7 years), the incidence of carotid atherosclerosis was 36.2% [13]. The very high prevalence of APCA in the Bialystok PLUS population is an unfavourable phenomenon, which could be explained by a low level of health education and low compliance with the principles of a healthy lifestyle [37,38].

### Limitations of the Study 

Potential participants were randomly selected from the general population, but only those who responded to the invitation took part in the study. It can be speculated that people who are interested and concerned about their health may be more likely to participate in the study. On the other hand, people who are seriously ill, bedridden or have an unhealthy lifestyle may be underrepresented. This type of error is unavoidable in this study design. However, the deviation of the studied population from the available characteristics of the general population is marginal [39]. 

The FGCR2A is a cell surface protein, but we were not able to measure cell surface abundance, only semi-quantitative plasma concentrations. Many cell surface proteins undergo ectodomain shedding, a process in which part of the protein is released in a soluble form. This can be a mechanism to reduce membrane expression of a protein. The concentration of the biomarker in plasma is a result of the number of cells on which it occurs, its membrane expression and the shedding process, which are subject to further unknown regulatory processes. This makes it more difficult to determine the biological significance of the plasma concentration of such a biomarker and to interpret the results of our study.

Carotid ultrasound was not performed by a physician but by an experienced certified technician according to the standard operating procedures based on the widely accepted guidelines [13]. Next, multivariate logistic regression analyses were performed to identify biomarkers independently associated with APCA, but only 7 traditional risk factors for atherosclerosis were included in the models. Therefore, the current state of knowledge regarding the pathogenesis of APCA was not fully exploited. 

## 6. Conclusions

In the general population of the Bialystok PLUS, the prevalence of atherosclerotic plaques in the carotid arteries is very high. A wide range of biomarkers has been associated with APCA, including biomarkers involved in cellular metabolism, cell adhesion, immune response, and complement activation. However, most of these associations were explained by the interaction of biomarkers with traditional risk factors for atherosclerosis. The only biomarker that was independently associated with APCA was the FCGR2A. The rs1801274 polymorphism of the FCGR2A gene was associated with the diagnosis of APCA, but this could not explain the link between APCA and the FCGR2A because the high-risk allele was associated with lower FCGR2A levels. We have identified a potential pathogenic pathway that requires further investigation. The FCGR2A might potentially be used in clinical practice as a marker to assess the likelihood of CA, but further research in this area is needed to replicate the results and to develop a diagnostic model. At present, there is no data on the cause-and-effect relationship between the biomarker and the development of atherosclerosis, so treating it as a drug target is highly speculative.

## Figures and Tables

**Table 1:** Clinical characteristics of the study group according to the presence of atherosclerotic plaques in the carotid arteries (APCA).

	Patients with APCA (n = 320)	Patients without APCA (n = 373)	*p*-Value
Age (years)	62 (53–69)	38 (31–45)	<0.001
Gender, men (n, %)	145 (45.3%)	156 (41.8%)	0.356
Current smoking (n, %)	74 (23.1%)	73 (19.6%)	0.254
Hypertension (n, %)	206 (64.4%)	107 (28.6%)	<0.001
Diabetes (n, %)	70 (21.9%)	23 (6.2%)	<0.001
Hypercholesterolaemia (n, %)	273 (85.3%)	220 (60%)	<0.001
BP systolic (mmHg)	128.5 (116.62–140.25)	119.3 (108.5–130.5)	<0.001
BP diastolic (mmHg)	82 (76.5–88.75)	80 (73.5–86.5)	0.002
BMI (kg/m^2^)	27.8 (24.34–31.24)	25.05 (22.59–28.36)	<0.001
IMT (mm)	0.71 (0.63–0.8)	0.55 (0.51–0.62)	<0.001
Biochemistry			
Fasting glucose (mmol/L)	5.72 (5.39–6.17)	5.39 (5.06–5.72)	<0.001
LDL-cholesterol (mmol/L)	3.40 (2.59–4.03)	3.05 (2.47–3.54)	<0.001
HDL-cholesterol (mmol/L)	1.52 (1.26–1.84)	1.59 (1.32–1.89)	0.184
Triglycerides (mmol/L)	1.16 (0.9–1.64)	0.94 (0.68–1.38)	<0.001
hsCRP (mg/L)	0.83 (0.41–1.65)	0.59 (0.27–1.44)	<0.001
Interleukin-6 (pg/mL)	3.07 (2.09–4.12)	2.34 (1.83–3.51)	<0.001
Pharmacotherapy			
Beta-blockers	104 (32.5%)	25 (6.7%)	<0.001
ACE-inhibitors or sartans	100 (31.3%)	34 (9.1%)	<0.001
Diuretics	32 (10%)	10 (2.7%)	<0.001
Lipid-lowering drugs	80 (25%)	16 (4.3%)	<0.001
Antidiabetic drugs	34 (10.6%)	9 (2.4%)	<0.001
Antiplatelet or anticoagulant drugs (%)	65 (20.3%)	8 (2.1%)	<0.001

BP—blood pressure, BMI—body mass index, IMT—intima-media thickness, LDL—low-density lipoprotein, HDL—high-density lipoprotein, CRP—C-reactive protein, ACE—angiotensin-converting enzyme.

**Table 2:** Univariate and multivariate linear regression with FCGR2A as dependent variable and traditional atherosclerosis risk factors as independent variables.

Variable	β	*p*
Univariate analysis		
Age	0.15	<0.001
Male sex	0.07	0.055
Diabetes	0.09	0.02
Hypercholesterolaemia	0.04	0.26
Obesity	0.13	<0.001
Current smoking	−0.02	0.75
Hypertension	0.1	0.004
Multivariate analysis, R2 = 0.028		
Age	0.13	0.001
Male sex	0.1	0.01

**Table 3:** Prevalence of the rs1801274 genotypes according to the presence of atherosclerotic plaques in the carotid arteries (APCA). The difference was not significant (*p* = 0.37).

Genotype	Participants with APCA N = 310	Participants without APCA N = 365
AA	89 (28.7%)	123 (33.7%)
AG	157 (50.6%)	170 (46.6%)
GG	64 (20.6%)	72 (19.7%)

## Data Availability

The datasets used and/or analysed during the current study are available from the corresponding author upon reasonable request.

## Supplementary Materials

The following supporting information can be downloaded at https://www.mdpi.com/article/10.3390/jcm12206480/s1, Supplementary Table S1: Biomarkers included in the Olink Target 96 Cardiometabolic panel. Supplementary Table S2: Comparison of biomarker levels between patients with and without APCA (atherosclerotic plaques in the carotid arteries). Univariate logistic regression for association between APCA (dependent variable) and biomarkers (independent variable). Supplementary Materials S1: 54 models of multivariate logistic regression with the presence of APCA (atherosclerotic plaques in the carotid arteries) as the dependent variable and one of the 54 biomarkers together with 7 risk factors for atherosclerosis as independent variables.
