# Supplementary material for: The FCGR2A Is Associated with the Presence of Atherosclerotic Plaques in the Carotid Arteries—A Case-Control Study
Source: J Clin Med. 2023 Oct 12;12(20):6480. doi: 10.3390/jcm12206480 (PMC10607679; doi:10.3390/jcm12206480)
Supplement: Supplementary file 1 [file jcm-12-06480-s001.zip › Supplementary material S1.pdf]

Supplementary material S1

54 models of multivariate logistic regression with the presence of APCA (atherosclerotic plaques in the carotid arteries) as the dependent variable and one of the 54 biomarkers together with 7 risk factors for atherosclerosis as independent variables.

ANGPTL3_OID01306

Logistic regression

	LR chi2(8)	=	401.95
	Prob > chi2	=	0.0000
Log likelihood = -259.33788	Pseudo R2	=	0.4366

APCA	Odds Ratio	Std. Err.	z	P>|z|	[95% Conf.	Interval]
ANGPTL3_OID01306	.9120918	.2683734	-0.31	0.754	.5123674	1.623662
age	1.162871	.0146405	11.99	0.000	1.134527	1.191922
male sex	2.370586	.5888668	3.47	0.001	1.456842	3.857438
diabetes	.7946559	.2795058	-0.65	0.513	.3988269	1.583339
hypercholesterolemia	1.013708	.2918634	0.05	0.962	.5765512	1.782329
obesity	1.081218	.2915088	0.29	0.772	.6374113	1.834033
current smoking	2.026633	.5519487	2.59	0.009	1.188372	3.456192
hypertension	1.067643	.2631674	0.27	0.791	.6585827	1.730781
_cons	.0004995	.0008891	-4.27	0.000	.0000153	.0163521

AOC3_OID01294

Logistic regression

	LR chi2(8)	=	401.95
	Prob > chi2	=	0.0000
Log likelihood = -259.33792	Pseudo R2	=	0.4366

APCA	Odds Ratio	Std. Err.	z	P>|z|	[95% Conf.	Interval]
AOC3_OID01294	1.119541	.4043912	0.31	0.755	.5515391	2.2725
age	1.16092	.0144473	11.99	0.000	1.132946	1.189584
male sex	2.416032	.5918039	3.60	0.000	1.494865	3.904841
diabetes	.7939203	.2787152	-0.66	0.511	.3989812	1.579797
hypercholesterolemia	1.010329	.291061	0.04	0.972	.5744395	1.776976
obesity	1.074015	.2884054	0.27	0.790	.6345081	1.817957
current smoking	1.990678	.5429463	2.52	0.012	1.166381	3.397516
hypertension	1.070647	.2641247	0.28	0.782	.6601738	1.73634
_cons	.0001935	.00029	-5.70	0.000	.0000102	.0036521

C1QTNF1_OID01301

Logistic regression

LR chi2(8) = 401.98
Prob > chi2 = 0.0000
Log likelihood = -259.32331 Pseudo R2 = 0.4366

APCA	Odds Ratio	Std. Err.	z	P>|z|	[95% Conf.	Interval]
C1QTNF1_OID01301	.9005416	.2657176	-0.36	0.723	.505062	1.605694
age	1.162266	.0142683	12.25	0.000	1.134634	1.19057
male sex	2.424232	.5957944	3.60	0.000	1.497537	3.924378
diabetes	.8081635	.2859814	-0.60	0.547	.4039133	1.617001
hypercholesterolemia	.9961492	.28964	-0.01	0.989	.5634162	1.761244
obesity	1.084633	.2928547	0.30	0.763	.638933	1.841239
current smoking	2.028771	.552434	2.60	0.009	1.189737	3.459514
hypertension	1.075116	.2651963	0.29	0.769	.6629665	1.743489

```
                   _cons |    .000486   .0007403    -5.01   0.000     .0000246    .0096194
------------------------------------------------------------------------------
```

C2_OID01233

```
Logistic regression
                                                  LR chi2(8)        =     402.05
                                                  Prob > chi2       =     0.0000
Log likelihood = -259.28429                       Pseudo R2         =     0.4367

------------------------------------------------------------------------------
                    APCA | Odds Ratio   Std. Err.      z    P>|z|     [95% Conf. Interval]
-------------------------+----------------------------------------------------
             C2_OID01233 |   1.125049   .2927497     0.45   0.651     .6755847    1.873541
                     age |   1.161668   .0141943    12.26   0.000     1.134178    1.189824
                male sex |   2.389266   .5844112     3.56   0.000     1.479318    3.858933
                diabetes |   .7891884    .277238    -0.67   0.500     .3964221    1.571099
   hypercholesterolemia |   1.003863   .2896774     0.01   0.989     .5702289    1.767256
                 obesity |   1.063444   .2862012     0.23   0.819     .6275286     1.80217
         current smoking |   2.015402   .5465699     2.58   0.010     1.184456    3.429293
            hypertension |   1.065167   .2628293     0.26   0.798     .6567259    1.727632
                   _cons |   .0001309   .0002502    -4.68   0.000     3.09e-06    .0055406
------------------------------------------------------------------------------
```

CA3_OID01235

```
Logistic regression
                                                  LR chi2(8)        =     402.11
                                                  Prob > chi2       =     0.0000
Log likelihood = -259.25464                       Pseudo R2         =     0.4368

------------------------------------------------------------------------------
```

APCA	Odds Ratio	Std. Err.	z	P>|z|	[95% Conf.	Interval]
CA3_OID01235	.8411416	.2841723	-0.51	0.609	.4338086	1.630948
age	1.164068	.014984	11.80	0.000	1.135067	1.193809
male sex	2.465212	.6154464	3.61	0.000	1.511296	4.021229
diabetes	.8095897	.2854679	-0.60	0.549	.4056247	1.615867
hypercholesterolemia	.9930307	.2883556	-0.02	0.981	.5620712	1.754422
obesity	1.09262	.2955413	0.33	0.743	.6430263	1.856562
current smoking	2.024381	.54929	2.60	0.009	1.189404	3.445521
hypertension	1.066855	.2631229	0.26	0.793	.6579155	1.729979
_cons	.0003619	.0002636	-10.88	0.000	.0000868	.0015089

CCL18_OID01276

Logistic regression

LR chi2(8) = 402.91
Prob > chi2 = 0.0000
Pseudo R2 = 0.4377

Log likelihood = -258.85334

APCA	Odds Ratio	Std. Err.	z	P>|z|	[95% Conf.	Interval]
CCL18_OID01276	.857858	.1279692	-1.03	0.304	.6403821	1.149189
age	1.164743	.0146343	12.14	0.000	1.136411	1.193782
male sex	2.514091	.6256711	3.70	0.000	1.54364	4.094641
diabetes	.8018234	.2818553	-0.63	0.530	.4025929	1.59695
hypercholesterolemia	1.021072	.2938848	0.07	0.942	.58085	1.794937
obesity	1.087856	.2928225	0.31	0.754	.6418742	1.84371
current smoking	2.022101	.550605	2.59	0.010	1.18584	3.448097
hypertension	1.068856	.2636811	0.27	0.787	.6590716	1.733429
_cons	.0007049	.0007279	-7.03	0.000	.0000931	.005335

CCL5_OID01246

Logistic regression

LR chi2(8) = 401.99

Prob > chi2 = 0.0000

Log likelihood = -259.3181 Pseudo R2 = 0.4366

APCA	Odds Ratio	Std. Err.	z	P>|z|	[95% Conf.	Interval]
CCL5_OID01246	.9422708	.1513577	-0.37	0.711	.6877752	1.290937
age	1.161227	.0142473	12.18	0.000	1.133636	1.189489
male sex	2.390182	.5855182	3.56	0.000	1.478815	3.863209
diabetes	.8013646	.2818184	-0.63	0.529	.4022401	1.596522
hypercholesterolemia	1.019245	.2948202	0.07	0.947	.5781832	1.796766
obesity	1.075013	.2888014	0.27	0.788	.6349498	1.820072
current smoking	2.018983	.5478224	2.59	0.010	1.186236	3.436324
hypertension	1.069849	.2637042	0.27	0.784	.6599519	1.734335
_cons	.0004388	.0005373	-6.31	0.000	.0000398	.0048372

CD46_OID01251

Logistic regression

LR chi2(8) = 402.94

Prob > chi2 = 0.0000

Log likelihood = -258.83897 Pseudo R2 = 0.4377

APCA	Odds Ratio	Std. Err.	z	P>|z|	[95% Conf.	Interval]
CD46_OID01251	.623017	.282696	-1.04	0.297	.2560144	1.516126
age	1.164497	.0145387	12.20	0.000	1.136347	1.193343
male sex	2.4548	.6032966	3.65	0.000	1.516433	3.973829
diabetes	.8055766	.2831538	-0.62	0.538	.4044978	1.604344
hypercholesterolemia	.9928815	.2861754	-0.02	0.980	.5643624	1.746774
obesity	1.121373	.3048535	0.42	0.673	.65818	1.910537
current smoking	2.043494	.5552919	2.63	0.009	1.199695	3.480774
hypertension	1.054992	.2607304	0.22	0.829	.6499549	1.712438
_cons	.0017265	.0030822	-3.56	0.000	.0000522	.057121

CD59_OID01248

Logistic regression

LR chi2(8) = 402.72
Prob > chi2 = 0.0000
Log likelihood = -258.94868 Pseudo R2 = 0.4374

APCA	Odds Ratio	Std. Err.	z	P>|z|	[95% Conf.	Interval]
CD59_OID01248	.6634322	.2883562	-0.94	0.345	.2830269	1.555125
age	1.164872	.0147119	12.08	0.000	1.136391	1.194066
male sex	2.546197	.6437789	3.70	0.000	1.551225	4.179355
diabetes	.7830771	.2758397	-0.69	0.488	.3926159	1.561857
hypercholesterolemia	.9981043	.2878598	-0.01	0.995	.5671316	1.75658
obesity	1.141777	.3159394	0.48	0.632	.6638144	1.963885
current smoking	2.034601	.5537307	2.61	0.009	1.19349	3.468483
hypertension	1.077851	.2660555	0.30	0.761	.6644298	1.748511

_cons	.0003772	.0002506	-11.86	0.000	.0001026	.001387

CDH1_OID01245

Logistic regression

LR chi2(8) = 403.19

Prob > chi2 = 0.0000

Log likelihood = -258.71783   Pseudo R2 = 0.4379

APCA	Odds Ratio	Std. Err.	z	P>|z|	[95% Conf.	Interval]
CDH1_OID01245	.6983075	.2203616	-1.14	0.255	.3762158	1.296153
age	1.163715	.0143659	12.28	0.000	1.135897	1.192215
male sex	2.484688	.6137814	3.68	0.000	1.531099	4.032184
diabetes	.8487994	.3027783	-0.46	0.646	.4218615	1.707813
hypercholesterolemia	1.000914	.2885938	0.00	0.997	.5688134	1.761262
obesity	1.102647	.2979672	0.36	0.718	.6492577	1.872647
current smoking	2.079438	.5689532	2.68	0.007	1.216324	3.555024
hypertension	1.056563	.2610238	0.22	0.824	.6510376	1.714686
_cons	.0011304	.0014848	-5.17	0.000	.0000861	.0148375

CES1_OID01263

Logistic regression

LR chi2(8) = 402.08

Prob > chi2 = 0.0000

Log likelihood = -259.27152   Pseudo R2 = 0.4367

APCA	Odds Ratio	Std. Err.	z	P>|z|	[95% Conf.	Interval]
CES1_OID01263	1.079356	.1713971	0.48	0.631	.7906746	1.473438
age	1.161929	.0142064	12.28	0.000	1.134416	1.190109
male sex	2.364765	.5830628	3.49	0.000	1.458524	3.834092
diabetes	.7798669	.2753864	-0.70	0.481	.3903412	1.558104
hypercholesterolemia	.9964165	.2887253	-0.01	0.990	.5646686	1.758281
obesity	1.040417	.2871882	0.14	0.886	.6056879	1.787171
current smoking	1.987122	.5404762	2.52	0.012	1.166022	3.386432
hypertension	1.053694	.2620482	0.21	0.833	.6471803	1.715552
_cons	.0002296	.0001882	-10.22	0.000	.0000461	.0011445

CHL1_OID01216

Logistic regression

	LR chi2(8)	=	402.52
	Prob > chi2	=	0.0000
Log likelihood = -259.04853	Pseudo R2	=	0.4372

APCA	Odds Ratio	Std. Err.	z	P>|z|	[95% Conf.	Interval]
CHL1_OID01216	1.350669	.4964934	0.82	0.413	.6571359	2.776149
age	1.160849	.0142014	12.19	0.000	1.133346	1.18902
male sex	2.373512	.5803879	3.53	0.000	1.469771	3.832951
diabetes	.8192509	.2888823	-0.57	0.572	.4104576	1.63518
hypercholesterolemia	1.006487	.2899212	0.02	0.982	.5722912	1.770105
obesity	1.08257	.2910229	0.30	0.768	.6391916	1.833501
current smoking	1.967975	.5356518	2.49	0.013	1.154346	3.355083
hypertension	1.073829	.2653251	0.29	0.773	.6616334	1.742821
_cons	.0000878	.0001424	-5.76	0.000	3.65e-06	.0021114

CNDP1_OID01299

Logistic regression

	LR chi2(8)	=	402.94
	Prob > chi2	=	0.0000
Log likelihood = -258.84139	Pseudo R2	=	0.4377

APCA	Odds Ratio	Std. Err.	z	P>|z|	[95% Conf.	Interval]
CNDP1_OID01299	.7789438	.1873618	-1.04	0.299	.4861429	1.248097
age	1.160146	.0142467	12.10	0.000	1.132556	1.188408
male sex	2.463013	.6068639	3.66	0.000	1.519632	3.992041
diabetes	.8024985	.2819098	-0.63	0.531	.4031118	1.597581
hypercholesterolemia	1.033088	.298853	0.11	0.910	.5860044	1.821269
obesity	1.133144	.3097574	0.46	0.647	.663132	1.93629
current smoking	2.010781	.5451903	2.58	0.010	1.181885	3.421008
hypertension	1.07894	.2661375	0.31	0.758	.6653267	1.749683
_cons	.0013591	.0021453	-4.18	0.000	.0000616	.0299793

COL18A1_OID01271

Logistic regression

	LR chi2(8)	=	401.90
	Prob > chi2	=	0.0000
Log likelihood = -259.36309	Pseudo R2	=	0.4365

APCA	Odds Ratio	Std. Err.	z	P>|z|	[95% Conf.	Interval]
COL18A1_OID01271	.92961	.3115059	-0.22	0.828	.4820244	1.792803
age	1.162126	.0142819	12.23	0.000	1.134468	1.190458
male sex	2.418063	.5949575	3.59	0.000	1.492904	3.916547
diabetes	.8002681	.2814369	-0.63	0.526	.4016858	1.594353
hypercholesterolemia	1.010085	.2907614	0.03	0.972	.5745563	1.775757
obesity	1.075309	.288872	0.27	0.787	.6351347	1.820543
current smoking	2.013675	.5461454	2.58	0.010	1.183391	3.4265
hypertension	1.07298	.2648176	0.29	0.775	.66147	1.740496
_cons	.0003871	.0005326	-5.71	0.000	.0000261	.0057407

COMP_OID01274

Logistic regression

LR chi2(8) = 401.98
Prob > chi2 = 0.0000
Pseudo R2 = 0.4366

Log likelihood = -259.32266

APCA	Odds Ratio	Std. Err.	z	P>|z|	[95% Conf.	Interval]
COMP_OID01274	1.102269	.2996027	0.36	0.720	.6470332	1.877798
age	1.160776	.014452	11.97	0.000	1.132793	1.18945
male sex	2.363141	.5876902	3.46	0.001	1.451457	3.84747
diabetes	.7951646	.2788616	-0.65	0.513	.3998926	1.581141
hypercholesterolemia	1.011571	.2916072	0.04	0.968	.5749353	1.77981
obesity	1.052643	.2885461	0.19	0.852	.6151104	1.801395
current smoking	2.00044	.5421742	2.56	0.011	1.176052	3.402707
hypertension	1.071808	.2642819	0.28	0.779	.6610458	1.737812
_cons	.0001389	.0003065	-4.02	0.000	1.83e-06	.0105129

CR2_OID01258

Logistic regression

	LR chi2(8)	=	402.21
	Prob > chi2	=	0.0000
Log likelihood = -259.20475	Pseudo R2	=	0.4369

APCA	Odds Ratio	Std. Err.	z	P>|z|	[95% Conf.	Interval]
CR2_OID01258	.8673713	.2046541	-0.60	0.546	.5462182	1.377349
age	1.159258	.0147044	11.65	0.000	1.130793	1.188439
male sex	2.440158	.5998004	3.63	0.000	1.507264	3.95045
diabetes	.8061433	.283715	-0.61	0.540	.4044263	1.606886
hypercholesterolemia	1.008863	.2900567	0.03	0.976	.5742542	1.772392
obesity	1.081281	.2903396	0.29	0.771	.6388202	1.8302
current smoking	1.986007	.5407742	2.52	0.012	1.164676	3.386541
hypertension	1.079091	.2662234	0.31	0.758	.6653613	1.750083
_cons	.0010061	.0021246	-3.27	0.001	.000016	.0631214

CRTAC1_OID01304

Logistic regression

	LR chi2(8)	=	403.74
	Prob > chi2	=	0.0000
Log likelihood = -258.44199	Pseudo R2	=	0.4385

APCA	Odds Ratio	Std. Err.	z	P>|z|	[95% Conf.	Interval]

CRTAC1_OID01304	1.379303	.3228022	1.37	0.169	.8718677	2.182071
age	1.156933	.0145331	11.60	0.000	1.128796	1.185771
male sex	2.466043	.6051593	3.68	0.000	1.524468	3.989173
diabetes	.8162426	.2871971	-0.58	0.564	.409564	1.626735
hypercholesterolemia	.9958105	.2874296	-0.01	0.988	.5655706	1.753342
obesity	1.094434	.2951692	0.33	0.738	.6450901	1.856774
current smoking	2.032729	.5499792	2.62	0.009	1.196125	3.454477
hypertension	1.060579	.2622628	0.24	0.812	.6532144	1.721989
_cons	.0001029	.0001035	-9.13	0.000	.0000143	.0007388

CST3_OID01225

Logistic regression

	LR chi2(8)	=	402.36
	Prob > chi2	=	0.0000
Log likelihood = -259.1332	Pseudo R2	=	0.4370

APCA	Odds Ratio	Std. Err.	z	P>|z|	[95% Conf.	Interval]
CST3_OID01225	.8013045	.249718	-0.71	0.477	.4350405	1.475929
age	1.164619	.0148495	11.95	0.000	1.135875	1.19409
male sex	2.474368	.6138479	3.65	0.000	1.521584	4.023766
diabetes	.7993626	.2809556	-0.64	0.524	.4013915	1.591913
hypercholesterolemia	1.000656	.2878912	0.00	0.998	.5693663	1.758642
obesity	1.117707	.3067077	0.41	0.685	.6527571	1.913835
current smoking	2.06154	.5643131	2.64	0.008	1.205561	3.525287
hypertension	1.077242	.2656989	0.30	0.763	.6643035	1.746867
_cons	.0011499	.0022953	-3.39	0.001	.000023	.0575127

DEFA1_OID01277

Logistic regression

LR chi2(8)	=	401.85
Prob > chi2	=	0.0000
Pseudo R2	=	0.4365

Log likelihood = -259.38368

APCA	Odds Ratio	Std. Err.	z	P>|z|	[95% Conf.	Interval]
DEFA1_OID01277	.9833891	.2092625	-0.08	0.937	.6480254	1.492309
age	1.161784	.0141955	12.27	0.000	1.134292	1.189943
male sex	2.404989	.5879861	3.59	0.000	1.489383	3.88347
diabetes	.7984884	.2810037	-0.64	0.523	.4006029	1.59156
hypercholesterolemia	1.009498	.2908864	0.03	0.974	.5738946	1.775738
obesity	1.073679	.2882367	0.26	0.791	.6344007	1.817128
current smoking	2.008136	.5443542	2.57	0.010	1.180468	3.416112
hypertension	1.069667	.2636142	0.27	0.785	.6598943	1.733896
_cons	.0003003	.0001939	-12.56	0.000	.0000847	.0010649

EFEMP1_OID01281

Logistic regression

LR chi2(8)	=	401.85
Prob > chi2	=	0.0000
Pseudo R2	=	0.4365

Log likelihood = -259.38556

APCA	Odds Ratio	Std. Err.	z	P>|z|	[95% Conf.	Interval]
EFEMP1_OID01281	1.015315	.3116572	0.05	0.961	.5563129	1.853029
age	1.161597	.0148074	11.75	0.000	1.132935	1.190985
male sex	2.405421	.5889316	3.58	0.000	1.488631	3.886826
diabetes	.796538	.2799179	-0.65	0.517	.4000174	1.586113
hypercholesterolemia	1.011606	.2929648	0.04	0.968	.5734561	1.784524
obesity	1.071182	.291568	0.25	0.801	.6283074	1.826224
current smoking	2.007917	.5448977	2.57	0.010	1.179645	3.41775
hypertension	1.070833	.264698	0.28	0.782	.6596509	1.738318
_cons	.0002741	.0004572	-4.92	0.000	.0000104	.0072072

F11_OID01227

Logistic regression

	LR chi2(8)	=	402.07
	Prob > chi2	=	0.0000
Log likelihood = -259.27746	Pseudo R2	=	0.4367

APCA	Odds Ratio	Std. Err.	z	P>|z|	[95% Conf.	Interval]
F11_OID01227	.844676	.3050498	-0.47	0.640	.4161824	1.714339
age	1.162333	.0142818	12.24	0.000	1.134676	1.190665
male sex	2.422696	.5944195	3.61	0.000	1.497796	3.918728
diabetes	.783925	.2769885	-0.69	0.491	.3922066	1.566874
hypercholesterolemia	1.025951	.297735	0.09	0.930	.5809042	1.81196
obesity	1.09283	.2967421	0.33	0.744	.6418317	1.860733
current smoking	2.006695	.5441364	2.57	0.010	1.179422	3.414236
hypertension	1.067877	.2632264	0.27	0.790	.6587252	1.731164
_cons	.0009604	.0024829	-2.69	0.007	6.05e-06	.1524292

FCGR2A_OID01244

Logistic regression

	LR chi2(8)	=	408.37
	Prob > chi2	=	0.0000
Log likelihood = -256.12477	Pseudo R2	=	0.4436

APCA	Odds Ratio	Std. Err.	z	P>|z|	[95% Conf.	Interval]
FCGR2A_OID01244	1.722756	.3623708	2.59	0.010	1.140719	2.601768
age	1.163612	.0144959	12.16	0.000	1.135545	1.192374
male sex	2.564405	.6354459	3.80	0.000	1.577842	4.167829
diabetes	.7569338	.265117	-0.80	0.427	.3809987	1.503808

hypercholesterolemia	1.005617	.289883	0.02	0.984	.5715598	1.769307
obesity	1.015346	.2751691	0.06	0.955	.5969384	1.727023
current smoking	2.024794	.5558104	2.57	0.010	1.18229	3.467668
hypertension	1.022635	.2556007	0.09	0.929	.6265686	1.669063
_cons	.000033	.0000359	-9.48	0.000	3.91e-06	.0002786

FETUB_OID01305

Logistic regression

LR chi2(8) = 403.48
Prob > chi2 = 0.0000
Log likelihood = -258.57039 Pseudo R2 = 0.4383

APCA	Odds Ratio	Std. Err.	z	P>|z|	[95% Conf.	Interval]
FETUB_OID01305	.7259676	.1825309	-1.27	0.203	.4435051	1.188327
age	1.160542	.0142095	12.16	0.000	1.133023	1.188729
male sex	2.341224	.5754329	3.46	0.001	1.446212	3.790127
diabetes	.7903415	.2790442	-0.67	0.505	.3956242	1.578871
hypercholesterolemia	1.027853	.2964951	0.10	0.924	.583973	1.809127
obesity	1.142822	.3129161	0.49	0.626	.6682074	1.954547
current smoking	2.026383	.5503836	2.60	0.009	1.189947	3.450765
hypertension	1.07821	.2661672	0.31	0.760	.6646232	1.749167
_cons	.000755	.0007126	-7.62	0.000	.0001187	.0048009

GP1BA_OID01234

Logistic regression

LR chi2(8) = 404.82

```
                                                  Prob > chi2       =     0.0000
Log likelihood = -257.89867                       Pseudo R2         =     0.4397
```

APCA	Odds Ratio	Std. Err.	z	P>|z|	[95% Conf.	Interval]
GP1BA_OID01234	.561618	.1879682	-1.72	0.085	.2914419	1.082256
age	1.161906	.0142642	12.22	0.000	1.134283	1.190203
male sex	2.497434	.6166722	3.71	0.000	1.539264	4.052048
diabetes	.7337331	.259904	-0.87	0.382	.3664581	1.469102
hypercholesterolemia	1.009698	.2922609	0.03	0.973	.5725426	1.780635
obesity	1.074499	.2893216	0.27	0.790	.6338845	1.821387
current smoking	2.02436	.5501262	2.60	0.009	1.188423	3.448295
hypertension	1.059839	.2623113	0.23	0.814	.652479	1.721524
_cons	.0099182	.0208603	-2.19	0.028	.0001607	.6119528

ICAM1_OID01230

```
Logistic regression
                                                  LR chi2(8)        =     402.28
                                                  Prob > chi2       =     0.0000
Log likelihood =  -259.1723                       Pseudo R2         =     0.4370
```

APCA	Odds Ratio	Std. Err.	z	P>|z|	[95% Conf.	Interval]
ICAM1_OID01230	1.241202	.4096016	0.65	0.513	.650044	2.369968
age	1.160849	.0142472	12.15	0.000	1.133258	1.189111

male sex	2.408673	.5885281	3.60	0.000	1.492099	3.888284
diabetes	.7836318	.2753494	-0.69	0.488	.3935684	1.560285
hypercholesterolemia	1.010133	.2917905	0.03	0.972	.5734531	1.779343
obesity	1.046048	.283904	0.17	0.868	.614512	1.780628
current smoking	1.943065	.5361059	2.41	0.016	1.131448	3.336876
hypertension	1.061059	.2620982	0.24	0.810	.653852	1.721867
_cons	.0000687	.0001598	-4.12	0.000	7.18e-07	.006567

ICAM3_OID01267

Logistic regression

LR chi2(8) = 401.92

Prob > chi2 = 0.0000

Log likelihood = -259.35052 Pseudo R2 = 0.4366

APCA	Odds Ratio	Std. Err.	z	P>|z|	[95% Conf.	Interval]
ICAM3_OID01267	1.111116	.4349644	0.27	0.788	.5158733	2.393184
age	1.161457	.0142416	12.21	0.000	1.133877	1.189708
male sex	2.385532	.5865724	3.54	0.000	1.47328	3.862647
diabetes	.7929179	.2788242	-0.66	0.509	.3980237	1.579601
hypercholesterolemia	1.013656	.292431	0.05	0.963	.5758727	1.784247
obesity	1.068013	.2876401	0.24	0.807	.6299826	1.810608
current smoking	2.008316	.5443067	2.57	0.010	1.180685	3.416097
hypertension	1.072045	.2643078	0.28	0.778	.6612306	1.738092
_cons	.0002044	.0003092	-5.61	0.000	.0000105	.0039667

IGFBP3_OID01255

Logistic regression

```
                                                  LR chi2(8)        =     401.87
                                                  Prob > chi2       =     0.0000
Log likelihood = -259.37441                       Pseudo R2         =     0.4365
```

APCA	Odds Ratio	Std. Err.	z	P>|z|	[95% Conf.	Interval]
IGFBP3_OID01255	1.049787	.3240148	0.16	0.875	.5732966	1.922308
age	1.16227	.0145132	12.04	0.000	1.13417	1.191066
male sex	2.418029	.5979849	3.57	0.000	1.489214	3.926141
diabetes	.7973145	.2798665	-0.65	0.519	.4007266	1.586394
hypercholesterolemia	1.008339	.2906266	0.03	0.977	.5731534	1.773955
obesity	1.070338	.2881042	0.25	0.801	.6315419	1.814011
current smoking	2.007327	.5439111	2.57	0.010	1.18025	3.413989
hypertension	1.068116	.2635183	0.27	0.789	.6585908	1.732292
_cons	.0002306	.0003931	-4.91	0.000	8.16e-06	.0065144

IGFBP6_OID01264

```
Logistic regression
                                                  LR chi2(8)        =     404.82
                                                  Prob > chi2       =     0.0000
Log likelihood = -257.89843                       Pseudo R2         =     0.4397
```

APCA	Odds Ratio	Std. Err.	z	P>|z|	[95% Conf.	Interval]
IGFBP6_OID01264	.5274767	.1965841	-1.72	0.086	.2540803	1.095054

	Odds Ratio	Std. Err.	z	P>|z|	[95% Conf.	Interval]
age	1.16787	.0149015	12.16	0.000	1.139026	1.197445
male sex	2.83713	.7500363	3.94	0.000	1.689864	4.763286
diabetes	.8069431	.2835684	-0.61	0.542	.4052487	1.606809
hypercholesterolemia	.973395	.2808717	-0.09	0.926	.5529377	1.713571
obesity	1.162	.3163096	0.55	0.581	.6815527	1.981129
current smoking	1.96788	.5382314	2.48	0.013	1.151299	3.363638
hypertension	1.038124	.2575885	0.15	0.880	.6383243	1.688328
_cons	.0114098	.0249644	-2.04	0.041	.0001566	.8311561

IGLC2_OID01240

Logistic regression

LR chi2(8) = 401.87
Prob > chi2 = 0.0000
Log likelihood = -259.37809 Pseudo R2 = 0.4365

APCA	Odds Ratio	Std. Err.	z	P>|z|	[95% Conf.	Interval]
IGLC2_OID01240	.9707055	.2189176	-0.13	0.895	.6239086	1.510268
age	1.162013	.0142836	12.22	0.000	1.134353	1.190349
male sex	2.411152	.5920384	3.58	0.000	1.490112	3.901486
diabetes	.8001317	.2819889	-0.63	0.527	.4010275	1.596426
hypercholesterolemia	1.006667	.2911355	0.02	0.982	.5710996	1.774433
obesity	1.077932	.2912763	0.28	0.781	.6347194	1.830632
current smoking	2.014276	.5468829	2.58	0.010	1.183082	3.42944
hypertension	1.069523	.2635601	0.27	0.785	.6598279	1.733604
_cons	.0003561	.0005458	-5.18	0.000	.0000177	.0071789

KIT_OID01241

Logistic regression

	LR chi2(8)	=	402.42
	Prob > chi2	=	0.0000
Log likelihood = -259.09897	Pseudo R2	=	0.4371

APCA	Odds Ratio	Std. Err.	z	P>|z|	[95% Conf.	Interval]
KIT_OID01241	.7667119	.2690311	-0.76	0.449	.385438	1.525141
age	1.160052	.0143212	12.03	0.000	1.132319	1.188463
male sex	2.405086	.588945	3.58	0.000	1.488307	3.886587
diabetes	.7834181	.2759429	-0.69	0.488	.3928035	1.562471
hypercholesterolemia	1.011462	.2909836	0.04	0.968	.5755339	1.777577
obesity	1.061828	.2856312	0.22	0.824	.6267311	1.798982
current smoking	2.06197	.5632658	2.65	0.008	1.207149	3.522119
hypertension	1.045557	.259662	0.18	0.858	.6426192	1.701148
_cons	.0012003	.0023266	-3.47	0.001	.0000269	.0536021

LILRB1_OID01297

Logistic regression

	LR chi2(8)	=	401.94
	Prob > chi2	=	0.0000
Log likelihood = -259.33954	Pseudo R2	=	0.4366

APCA	Odds Ratio	Std. Err.	z	P>|z|	[95% Conf.	Interval]
LILRB1_OID01297	1.122064	.4203317	0.31	0.759	.5384593	2.338206
age	1.161427	.0142404	12.21	0.000	1.133849	1.189676

male sex	2.409192	.5891039	3.60	0.000	1.491876	3.890542
diabetes	.7915828	.2783731	-0.66	0.506	.3973354	1.577013
hypercholesterolemia	1.014356	.2926897	0.05	0.961	.5762065	1.785674
obesity	1.061493	.2876523	0.22	0.826	.6240959	1.805439
current smoking	2.008443	.5441921	2.57	0.010	1.180931	3.415816
hypertension	1.069753	.263711	0.27	0.784	.6598555	1.734276
_cons	.0002125	.0002651	-6.78	0.000	.0000184	.0024517

LILRB2_OID01296

Logistic regression

LR chi2(8) = 401.94

Prob > chi2 = 0.0000

Log likelihood = -259.34202 Pseudo R2 = 0.4366

APCA	Odds Ratio	Std. Err.	z	P>|z|	[95% Conf.	Interval]
LILRB2_OID01296	1.098951	.3463934	0.30	0.765	.5924834	2.038357
age	1.1616	.0142178	12.24	0.000	1.134065	1.189803
male sex	2.399268	.586499	3.58	0.000	1.485947	3.873952
diabetes	.7926131	.2787308	-0.66	0.509	.3978572	1.579048
hypercholesterolemia	1.010303	.2913033	0.04	0.972	.5741457	1.777791
obesity	1.06265	.2878084	0.22	0.822	.6249578	1.806882
current smoking	2.015146	.5461676	2.59	0.010	1.184689	3.427747
hypertension	1.071036	.2641193	0.28	0.781	.6605355	1.736648
_cons	.0002047	.0002832	-6.14	0.000	.0000136	.0030812

MFAP5_OID01285

Logistic regression

LR chi2(8) = 403.48

Prob > chi2 = 0.0000
Log likelihood = -258.56918 Pseudo R2 = 0.4383

APCA	Odds Ratio	Std. Err.	z	P>|z|	[95% Conf.	Interval]
MFAP5_OID01285	1.723342	.7339535	1.28	0.201	.7479171	3.970904
age	1.160333	.0142536	12.11	0.000	1.13273	1.188608
male sex	2.354436	.5771906	3.49	0.000	1.456179	3.806792
diabetes	.7882844	.2767774	-0.68	0.498	.3961088	1.568741
hypercholesterolemia	1.04632	.3047882	0.16	0.876	.5911714	1.851892
obesity	1.010972	.2762039	0.04	0.968	.5918147	1.727002
current smoking	2.024136	.5477934	2.61	0.009	1.190909	3.440337
hypertension	1.099347	.2717807	0.38	0.702	.6771749	1.784716
_cons	.0000991	.0001067	-8.56	0.000	.000012	.0008175

NRP1_OID01217
Logistic regression

LR chi2(8) = 404.37
Prob > chi2 = 0.0000
Log likelihood = -258.12606 Pseudo R2 = 0.4392

APCA	Odds Ratio	Std. Err.	z	P>|z|	[95% Conf.	Interval]
NRP1_OID01217	1.99495	.8699468	1.58	0.113	.8486818	4.689422
age	1.16063	.0142248	12.15	0.000	1.133083	1.188848
male sex	2.327184	.5715723	3.44	0.001	1.438036	3.766098

diabetes	.7768151	.2740275	-0.72	0.474	.3890898	1.550906
hypercholesterolemia	1.056029	.3059152	0.19	0.851	.5985436	1.863183
obesity	1.062692	.2870129	0.23	0.822	.6259135	1.804267
current smoking	2.033338	.5520784	2.61	0.009	1.194254	3.461961
hypertension	1.082535	.2681595	0.32	0.749	.6661728	1.759125
_cons	.000108	.0000979	-10.08	0.000	.0000183	.000638

OSMR_OID01300

Logistic regression

LR chi2(8) = 402.29
Prob > chi2 = 0.0000
Log likelihood = -259.16584 Pseudo R2 = 0.4370

APCA	Odds Ratio	Std. Err.	z	P>|z|	[95% Conf.	Interval]
OSMR_OID01300	1.409023	.7271497	0.66	0.506	.5124378	3.874314
age	1.160642	.014274	12.11	0.000	1.133	1.188958
male sex	2.416689	.5907005	3.61	0.000	1.496806	3.901901
diabetes	.7867384	.2760936	-0.68	0.494	.3954708	1.565115
hypercholesterolemia	.9997504	.289092	-0.00	0.999	.567224	1.762092
obesity	1.068547	.2870319	0.25	0.805	.6311681	1.809016
current smoking	1.99136	.5400426	2.54	0.011	1.170335	3.38836
hypertension	1.071171	.2641215	0.28	0.780	.6606565	1.736768
_cons	.0002237	.0001692	-11.11	0.000	.0000508	.0009853

PAM_OID01256

Logistic regression

LR chi2(8)	= 402.10
Prob > chi2	= 0.0000
Pseudo R2	= 0.4368

Log likelihood = -259.25911

APCA	Odds Ratio	Std. Err.	z	P>|z|	[95% Conf.	Interval]
PAM_OID01256	.8317078	.3033905	-0.51	0.613	.4068829	1.700091
age	1.162164	.0142097	12.29	0.000	1.134644	1.190351
male sex	2.420023	.5928098	3.61	0.000	1.4973	3.911381
diabetes	.802558	.2822495	-0.63	0.532	.4028279	1.598944
hypercholesterolemia	1.006828	.2900166	0.02	0.981	.5724888	1.770695
obesity	1.078917	.2900891	0.28	0.778	.6369785	1.827474
current smoking	2.010865	.5455761	2.57	0.010	1.181517	3.422362
hypertension	1.078681	.2662723	0.31	0.759	.6649267	1.749895
_cons	.0004653	.0005034	-7.09	0.000	.0000558	.003878

PCOLCE_OID01289

Logistic regression

LR chi2(8)	= 402.84
Prob > chi2	= 0.0000
Pseudo R2	= 0.4376

Log likelihood = -258.89285

APCA	Odds Ratio	Std. Err.	z	P>|z|	[95% Conf.	Interval]

PCOLCE_OID01289	.7818545	.1938873	-0.99	0.321	.4808854	1.271189
age	1.165573	.0148625	12.02	0.000	1.136804	1.19507
male sex	2.53114	.6357559	3.70	0.000	1.547094	4.141099
diabetes	.8168688	.2874156	-0.57	0.565	.40988	1.627976
hypercholesterolemia	1.013314	.2930893	0.05	0.964	.5748356	1.786258
obesity	1.066078	.2861342	0.24	0.812	.6299805	1.804058
current smoking	2.067743	.5647037	2.66	0.008	1.210687	3.531514
hypertension	1.084295	.2678296	0.33	0.743	.6681806	1.759548
_cons	.0011571	.0017275	-4.53	0.000	.000062	.021585

PLA2G7_OID01283

Logistic regression

LR chi2(8) = 404.76
Prob > chi2 = 0.0000
Log likelihood = -257.93117 Pseudo R2 = 0.4397

APCA	Odds Ratio	Std. Err.	z	P>|z|	[95% Conf.	Interval]
PLA2G7_OID01283	1.891622	.7079874	1.70	0.089	.9083442	3.939291
age	1.162199	.0143034	12.21	0.000	1.1345	1.190574
male sex	2.225133	.5529727	3.22	0.001	1.367169	3.621512
diabetes	.8343156	.2931121	-0.52	0.606	.4190693	1.66102
hypercholesterolemia	.9192729	.2724514	-0.28	0.776	.5142424	1.643316
obesity	1.048694	.2839605	0.18	0.861	.6168281	1.782925
current smoking	2.007362	.5447272	2.57	0.010	1.179342	3.416739
hypertension	1.103936	.2741257	0.40	0.690	.6785414	1.796021
_cons	.0000669	.0000734	-8.75	0.000	7.77e-06	.0005754

PLXNB2_OID01218

Logistic regression

	LR chi2(8)	=	401.87
	Prob > chi2	=	0.0000
Log likelihood = -259.37737	Pseudo R2	=	0.4365

APCA	Odds Ratio	Std. Err.	z	P>|z|	[95% Conf.	Interval]
PLXNB2_OID01218	1.074377	.5617317	0.14	0.891	.3855767	2.99366
age	1.161507	.0143509	12.12	0.000	1.133717	1.189977
male sex	2.398062	.587404	3.57	0.000	1.483744	3.875804
diabetes	.79238	.2801353	-0.66	0.510	.3962805	1.584398
hypercholesterolemia	1.009429	.2908092	0.03	0.974	.5739194	1.775419
obesity	1.070484	.2882809	0.25	0.800	.6314693	1.814715
current smoking	2.002683	.5447967	2.55	0.011	1.175052	3.413245
hypertension	1.070209	.2637476	0.28	0.783	.6602286	1.734774
_cons	.0002569	.0003097	-6.86	0.000	.0000242	.0027275

PRCP_OID01272

Logistic regression

	LR chi2(8)	=	401.86
	Prob > chi2	=	0.0000
Log likelihood = -259.38229	Pseudo R2	=	0.4365

APCA	Odds Ratio	Std. Err.	z	P>|z|	[95% Conf.	Interval]
PRCP_OID01272	.9640072	.3731379	-0.09	0.925	.4514462	2.058517

age	1.161912	.0142408	12.24	0.000	1.134333	1.190162
male sex	2.418755	.612551	3.49	0.000	1.472396	3.973371
diabetes	.8013657	.2851095	-0.62	0.534	.3990163	1.609425
hypercholesterolemia	1.012335	.2924746	0.04	0.966	.5746493	1.783387
obesity	1.078041	.2933309	0.28	0.782	.6324504	1.837571
current smoking	2.014054	.5478352	2.57	0.010	1.181786	3.432442
hypertension	1.072035	.2651714	0.28	0.779	.660179	1.740831
_cons	.0003103	.0002468	-10.16	0.000	.0000653	.0014753

PROC_OID01228

Logistic regression

LR chi2(8) = 401.87

Prob > chi2 = 0.0000

Log likelihood = -259.37608 Pseudo R2 = 0.4365

APCA	Odds Ratio	Std. Err.	z	P>|z|	[95% Conf.	Interval]
PROC_OID01228	1.042201	.2943394	0.15	0.884	.5991761	1.812795
age	1.161764	.0141947	12.27	0.000	1.134273	1.189921
male sex	2.393566	.5886868	3.55	0.000	1.478074	3.876097
diabetes	.7965935	.2797242	-0.65	0.517	.4002552	1.585392
hypercholesterolemia	1.004287	.2920104	0.01	0.988	.5680143	1.775646
obesity	1.071828	.2880307	0.26	0.796	.6329699	1.814961
current smoking	2.006431	.5437832	2.57	0.010	1.179591	3.412848
hypertension	1.067563	.2635399	0.26	0.791	.6580586	1.731897
_cons	.0002405	.0003726	-5.38	0.000	.0000115	.0050084

PRSS2_OID01236

Logistic regression

LR chi2(8) = 401.88
Prob > chi2 = 0.0000
Log likelihood = -259.36933 Pseudo R2 = 0.4365

APCA	Odds Ratio	Std. Err.	z	P>|z|	[95% Conf.	Interval]
PRSS2_OID01236	.9610516	.2035674	-0.19	0.851	.6345232	1.455613
age	1.162102	.0142911	12.22	0.000	1.134427	1.190453
male sex	2.409651	.5898802	3.59	0.000	1.491355	3.893386
diabetes	.7966494	.2797738	-0.65	0.517	.4002537	1.58562
hypercholesterolemia	1.013887	.292697	0.05	0.962	.5757816	1.785341
obesity	1.075234	.2888455	0.27	0.787	.6350979	1.820395
current smoking	2.026446	.5566687	2.57	0.010	1.182792	3.471857
hypertension	1.071424	.264189	0.28	0.780	.6608068	1.737195
_cons	.0003271	.0002669	-9.84	0.000	.0000661	.0016187

PTPRS_OID01284
Logistic regression

LR chi2(8) = 403.12
Prob > chi2 = 0.0000
Log likelihood = -258.75003 Pseudo R2 = 0.4379

APCA	Odds Ratio	Std. Err.	z	P>|z|	[95% Conf.	Interval]
PTPRS_OID01284	.57253	.2839407	-1.12	0.261	.2165973	1.513364
age	1.161437	.0141726	12.26	0.000	1.133989	1.18955

male sex	2.359495	.5782407	3.50	0.000	1.459538	3.814369
diabetes	.8009636	.2822212	-0.63	0.529	.4015043	1.597848
hypercholesterolemia	1.000227	.2880452	0.00	0.999	.5688132	1.758845
obesity	1.072731	.2883565	0.26	0.794	.6334073	1.816766
current smoking	1.995544	.5419582	2.54	0.011	1.171895	3.398085
hypertension	1.05839	.2611926	0.23	0.818	.6525047	1.716752
_cons	.0007589	.0007795	-6.99	0.000	.0001014	.0056815

QPCT_OID01293

Logistic regression

LR chi2(8) = 402.84

Prob > chi2 = 0.0000

Log likelihood = -258.89257   Pseudo R2 = 0.4376

APCA	Odds Ratio	Std. Err.	z	P>|z|	[95% Conf.	Interval]
QPCT_OID01293	.6513743	.2815748	-0.99	0.321	.279174	1.5198
age	1.161393	.0141761	12.26	0.000	1.133938	1.189513
male sex	2.437709	.5973855	3.64	0.000	1.507948	3.940738
diabetes	.8103822	.2857144	-0.60	0.551	.4060541	1.61732
hypercholesterolemia	.9885269	.2852939	-0.04	0.968	.561471	1.740402
obesity	1.101955	.297546	0.36	0.719	.6491204	1.870692
current smoking	2.007307	.5444636	2.57	0.010	1.179596	3.415815
hypertension	1.063165	.2626441	0.25	0.804	.6551186	1.725366
_cons	.000463	.0003499	-10.16	0.000	.0001053	.002036

REG1A_OID01231

Logistic regression

	LR chi2(8)	=	402.19
	Prob > chi2	=	0.0000
Log likelihood = -259.21532	Pseudo R2	=	0.4369

APCA	Odds Ratio	Std. Err.	z	P>|z|	[95% Conf.	Interval]
REG1A_OID01231	1.127916	.2325073	0.58	0.559	.7530284	1.689439
age	1.160356	.0143696	12.01	0.000	1.132531	1.188864
male sex	2.397315	.5862501	3.58	0.000	1.48446	3.871522
diabetes	.7859922	.276644	-0.68	0.494	.3942962	1.566801
hypercholesterolemia	1.007251	.2906943	0.03	0.980	.5721101	1.773356
obesity	1.083416	.2912329	0.30	0.766	.639711	1.834875
current smoking	1.970902	.5382063	2.48	0.013	1.154044	3.365952
hypertension	1.073724	.2648995	0.29	0.773	.6620514	1.741379
_cons	.0001342	.000201	-5.95	0.000	7.13e-06	.0025261

SAA4_OID01269

Logistic regression

	LR chi2(8)	=	402.14
	Prob > chi2	=	0.0000
Log likelihood = -259.24006	Pseudo R2	=	0.4368

APCA	Odds Ratio	Std. Err.	z	P>|z|	[95% Conf.	Interval]
SAA4_OID01269	1.122028	.2390328	0.54	0.589	.7390378	1.703494

age	1.161568	.0142015	12.25	0.000	1.134065	1.189739
male sex	2.442041	.6013972	3.63	0.000	1.507055	3.957099
diabetes	.7923971	.2779761	-0.66	0.507	.398417	1.57597
hypercholesterolemia	.9838888	.2875646	-0.06	0.956	.5548332	1.744736
obesity	1.048067	.2849047	0.17	0.863	.6151765	1.785575
current smoking	1.991038	.5411143	2.53	0.011	1.168811	3.391679
hypertension	1.065385	.2628208	0.26	0.797	.6569357	1.727787
_cons	.0001739	.0002036	-7.40	0.000	.0000175	.0017242

SELL_OID01249

Logistic regression

LR chi2(8) = 401.85
Prob > chi2 = 0.0000
Log likelihood = -259.38553 Pseudo R2 = 0.4365

APCA	Odds Ratio	Std. Err.	z	P>|z|	[95% Conf.	Interval]
SELL_OID01249	.9839626	.3176512	-0.05	0.960	.5226205	1.852554
age	1.161672	.0144442	12.05	0.000	1.133704	1.19033
male sex	2.402021	.5879085	3.58	0.000	1.486758	3.880728
diabetes	.7961476	.2802019	-0.65	0.517	.3994069	1.58698
hypercholesterolemia	1.009889	.2909495	0.03	0.973	.5741721	1.776255
obesity	1.074089	.2885749	0.27	0.790	.6343784	1.818579
current smoking	2.006503	.5468927	2.56	0.011	1.176078	3.423287
hypertension	1.069704	.2636218	0.27	0.785	.659919	1.733952
_cons	.0003452	.0010814	-2.54	0.011	7.44e-07	.1602081

SPARCL1_OID01287

Logistic regression

	LR chi2(8)	=	401.90
	Prob > chi2	=	0.0000
Log likelihood = -259.36204	Pseudo R2	=	0.4366

APCA	Odds Ratio	Std. Err.	z	P>|z|	[95% Conf.	Interval]
SPARCL1_OID01287	.918084	.3527529	-0.22	0.824	.4323421	1.949563
age	1.162417	.0144909	12.07	0.000	1.134359	1.191168
male sex	2.409153	.5896147	3.59	0.000	1.49122	3.892127
diabetes	.7960727	.2797353	-0.65	0.516	.3998025	1.585112
hypercholesterolemia	1.009255	.2907382	0.03	0.974	.5738432	1.77504
obesity	1.073509	.2881695	0.26	0.792	.6343251	1.816769
current smoking	2.009762	.5444113	2.58	0.010	1.181866	3.417599
hypertension	1.064604	.2634296	0.25	0.800	.6554864	1.72907
_cons	.0003939	.0005608	-5.51	0.000	.0000242	.0064157

TCN2_OID01259

Logistic regression

	LR chi2(8)	=	402.57
	Prob > chi2	=	0.0000
Log likelihood = -259.02707	Pseudo R2	=	0.4373

APCA	Odds Ratio	Std. Err.	z	P>|z|	[95% Conf.	Interval]
TCN2_OID01259	1.335452	.4553946	0.85	0.396	.6844866	2.605502
age	1.161234	.0142193	12.21	0.000	1.133696	1.18944

male sex	2.435901	.5963647	3.64	0.000	1.507529	3.935985
diabetes	.7751091	.2728729	-0.72	0.469	.3887783	1.545339
hypercholesterolemia	1.018309	.2928185	0.06	0.950	.5795798	1.789145
obesity	1.071099	.2874578	0.26	0.798	.6329759	1.812475
current smoking	1.958343	.5345996	2.46	0.014	1.146893	3.34391
hypertension	1.068773	.2637213	0.27	0.788	.6589464	1.733487
_cons	.000068	.0001261	-5.17	0.000	1.79e-06	.0025799

THBS4_OID01268

Logistic regression

LR chi2(8) = 401.96
Prob > chi2 = 0.0000
Log likelihood = -259.33133 Pseudo R2 = 0.4366

APCA	Odds Ratio	Std. Err.	z	P>|z|	[95% Conf.	Interval]
THBS4_OID01268	.9501884	.145913	-0.33	0.739	.70323	1.283873
age	1.162469	.0143624	12.18	0.000	1.134657	1.190962
male sex	2.40752	.5887435	3.59	0.000	1.490781	3.887995
diabetes	.8057979	.2846599	-0.61	0.541	.4032057	1.61037
hypercholesterolemia	1.001214	.2896392	0.00	0.997	.5679164	1.7651
obesity	1.090417	.2972965	0.32	0.751	.6390239	1.860666
current smoking	2.006605	.5438143	2.57	0.010	1.179712	3.413091
hypertension	1.074081	.2649617	0.29	0.772	.6623034	1.741877
_cons	.0003781	.0003623	-8.22	0.000	.0000578	.0024729

TIMD4_OID01298

Logistic regression

LR chi2(8)	=	403.73
Prob > chi2	=	0.0000
Log likelihood = -258.44415 Pseudo R2	=	0.4385

APCA	Odds Ratio	Std. Err.	z	P>|z|	[95% Conf.	Interval]
TIMD4_OID01298	.6963696	.1839686	-1.37	0.171	.4149229	1.168725
age	1.163849	.014325	12.33	0.000	1.136109	1.192267
male sex	2.353066	.5757768	3.50	0.000	1.456639	3.801163
diabetes	.7845562	.2763887	-0.69	0.491	.3933301	1.564916
hypercholesterolemia	1.008585	.2896752	0.03	0.976	.5744332	1.770866
obesity	1.154775	.3167169	0.52	0.600	.6745914	1.97676
current smoking	2.086283	.5686778	2.70	0.007	1.222794	3.559535
hypertension	1.088447	.2687729	0.34	0.731	.6708384	1.766024
_cons	.0016224	.0022166	-4.70	0.000	.0001115	.0236112

TIMP1_OID01224

Logistic regression

LR chi2(8)	=	401.95
Prob > chi2	=	0.0000
Log likelihood = -259.33377 Pseudo R2	=	0.4366

APCA	Odds Ratio	Std. Err.	z	P>|z|	[95% Conf.	Interval]
TIMP1_OID01224	1.136403	.4467167	0.33	0.745	.5259292	2.455484
age	1.161598	.0142112	12.24	0.000	1.134076	1.189788

male sex	2.395819	.585952	3.57	0.000	1.483451	3.86932
diabetes	.7950003	.2792509	-0.65	0.514	.3993698	1.582557
hypercholesterolemia	1.008313	.2904956	0.03	0.977	.5732759	1.773483
obesity	1.059906	.2875667	0.21	0.830	.6227665	1.803889
current smoking	1.99472	.542692	2.54	0.011	1.170309	3.399879
hypertension	1.067451	.2632926	0.26	0.791	.6582556	1.731018
_cons	.0001449	.0003313	-3.87	0.000	1.64e-06	.0127823

TNXB_OID01260

Logistic regression

LR chi2(8) = 401.98
Prob > chi2 = 0.0000
Log likelihood = -259.3214   Pseudo R2 = 0.4366

APCA	Odds Ratio	Std. Err.	z	P>|z|	[95% Conf.	Interval]
TNXB_OID01260	1.192221	.5793166	0.36	0.717	.4599874	3.090063
age	1.16221	.0142599	12.25	0.000	1.134594	1.190497
male sex	2.387893	.5848641	3.55	0.000	1.477512	3.859213
diabetes	.8006191	.2809935	-0.63	0.526	.40242	1.592841
hypercholesterolemia	1.010882	.2914452	0.04	0.970	.5745032	1.778724
obesity	1.067944	.2871672	0.24	0.807	.6304678	1.808982
current smoking	1.997349	.5423892	2.55	0.011	1.173022	3.40096
hypertension	1.081	.2682658	0.31	0.754	.6646414	1.758181
_cons	.0002006	.00025	-6.83	0.000	.0000174	.0023079

VCAM1_OID01257

Logistic regression

	LR chi2(8)	=	401.93
	Prob > chi2	=	0.0000
Log likelihood = -259.34583	Pseudo R2	=	0.4366

APCA	Odds Ratio	Std. Err.	z	P>|z|	[95% Conf.	Interval]
VCAM1_OID01257	.9050657	.315426	-0.29	0.775	.457116	1.791983
age	1.162281	.0143003	12.22	0.000	1.134588	1.19065
male sex	2.414073	.5912893	3.60	0.000	1.493695	3.901565
diabetes	.7998242	.2811668	-0.64	0.525	.4015751	1.593024
hypercholesterolemia	1.000521	.2899642	0.00	0.999	.5669392	1.765695
obesity	1.076477	.2890797	0.27	0.784	.6359475	1.822168
current smoking	2.007017	.5439583	2.57	0.010	1.179916	3.413899
hypertension	1.072939	.2646714	0.29	0.775	.6616095	1.739997
_cons	.0004857	.0008909	-4.16	0.000	.0000133	.0176851
