# Supplementary material for: The FCGR2A Is Associated with the Presence of Atherosclerotic Plaques in the Carotid Arteries—A Case-Control Study
Source: J Clin Med. 2023 Oct 12;12(20):6480. doi: 10.3390/jcm12206480 (PMC10607679; doi:10.3390/jcm12206480)
Supplement: Supplementary file 1 [file jcm-12-06480-s001.zip › Supplementary Table S1.pdf]

Supplementary Table S1:

Biomarkers included in the Olink Target 96 Cardiometabolic panel

Number	Abbreviation and a full name
1	ANG_OID01226 Angiogenin
2	ANGPTL3_OID01306 Angiopoietin-related protein 3
3	AOC3_OID01294 Membrane primary amine oxidase
4	APOM_OID01221 Apolipoprotein M
5	C1QTNF1_OID01301 Complement C1q tumor necrosis factor-related protein 1
6	C2_OID01233 Complement C2
7	CA1_OID01223 Carbonic anhydrase 1
8	CA3_OID01235 Carbonic anhydrase 3
9	CA4_OID01261 Carbonic anhydrase 4
10	CCL14_OID01292 C-C motif chemokine 14
11	CCL18_OID01276 C-C motif chemokine 18
12	CCL5_OID01246 C-C motif chemokine 5
13	CD46_OID01251 Membrane cofactor protein
14	CD59_OID01248 CD59 glycoprotein
15	CDH1_OID01245 Cadherin-1
16	CES1_OID01263 Liver carboxylesterase 1
17	CFHR5_OID01302 Complement factor H-related protein 5
18	CHL1_OID01216 Neural cell adhesion molecule L1-like protein
19	CNDP1_OID01299 Beta-Ala-His dipeptidase
20	COL18A1_OID01271 Collagen alpha-1(XVIII) chain
21	COMP_OID01274 Cartilage oligomeric matrix protein
22	CR2_OID01258 Complement receptor type 2
23	CRTAC1_OID01304 Cartilage acidic protein 1
24	CST3_OID01225 Cystatin-C
25	DEFA1_OID01277 Neutrophil defensin 1
26	DPP4_OID01266 Dipeptidyl peptidase 4
27	EFEMP1_OID01281 EGF-containing fibulin-like extracellular matrix protein 1
28	ENG_OID01254 FCGR3B_OID01219
29	F11_OID01227 Coagulation factor XI
30	F7_OID01239 Coagulation factor VII
31	FAP_OID01282 Prolyl endopeptidase FAP
32	FCGR2A_OID01244 Low affinity immunoglobulin gamma Fc region receptor II-a
33	FCGR3B_OID01219 Low affinity immunoglobulin gamma Fc region receptor III-B
34	FCN2_OID01290 Ficolin-2
35	FETUB_OID01305 Fetuin-B
36	GAS6_OID01286 Growth arrest-specific protein 6
37	GNLY_OID01262 Granulysin
38	GP1BA_OID01234 Platelet glycoprotein Ib alpha chain
39	ICAM1_OID01230 Intercellular adhesion molecule 1
40	ICAM3_OID01267 Intercellular adhesion molecule 3
41	IGFBP3_OID01255 Insulin-like growth factor-binding protein 3
42	IGFBP6_OID01264 Insulin-like growth factor-binding protein 6
43	IGLC2_OID01240 Ig lambda-2 chain C regions
44	IL7R_OID01253 Interleukin-7 receptor subunit alpha
45	ITGAM_OID01242 Integrin alpha-M

46	KIT_OID01241 Mast/stem cell growth factor receptor Kit
47	LCN2_OID01278 Neutrophil gelatinase-associated lipocalin
48	LILRB1_OID01297 Leukocyte immunoglobulin-like receptor subfamily B member 1
49	LILRB2_OID01296 Leukocyte immunoglobulin-like receptor subfamily B member 2
50	LILRB5_OID01220 Leukocyte immunoglobulin-like receptor subfamily B member 5
51	LTBP2_OID01288 Latent-transforming growth factor beta-binding protein 2
52	LYVE1_OID01307 Lymphatic vessel endothelial hyaluronic acid receptor 1
53	MBL2_OID01243 Mannose-binding protein C
54	MEGF9_OID01303 Multiple epidermal growth factor-like domains protein 9
55	MET_OID01238 Hepatocyte growth factor receptor
56	MFAP5_OID01285 Microfibrillar-associated protein 5
57	NCAM1_OID01247 Neural cell adhesion molecule 1
58	NID1_OID01250 Nidogen-1
59	NOTCH1_OID01273 Neurogenic locus notch homolog protein 1
60	NRP1_OID01217 Neuropilin-1
61	OSMR_OID01300 Oncostatin-M-specific receptor subunit beta
62	PAM_OID01256 Peptidyl-glycine alpha-amidating monooxygenase
63	PCOLCE_OID01289 Procollagen C-endopeptidase enhancer 1
64	PLA2G7_OID01283 Platelet-activating factor acetylhydrolase
65	PLTP_OID01275 Phospholipid transfer protein
66	PLXNB2_OID01218 Plexin-B2
67	PRCP_OID01272 Lysosomal Pro-X carboxypeptidase
68	PROC_OID01228 Vitamin K-dependent protein C
69	PRSS2_OID01236 Trypsin-2
70	PTPRS_OID01284 Receptor-type tyrosine-protein phosphatase S
71	QPCT_OID01293 Glutaminyl-peptide cyclotransferase
72	REG1A_OID01231 Lithostathine-1-alpha
73	REG3A_OID01280 Regenerating islet-derived protein 3-alpha
74	SAA4_OID01269 Serum amyloid A-4 protein
75	SELL_OID01249 L-selectin
76	SERPINA5_OID01229 Plasma serine protease inhibitor
77	SERPINA7_OID01232 Thyroxine-binding globulin
78	SOD1_OID01222 Superoxide dismutase [Cu-Zn]
79	SPARCL1_OID01287 SPARC-like protein 1
80	ST6GAL1_OID01252 Beta-galactoside alpha-2,6-sialyltransferase 1
81	TCN2_OID01259 Transcobalamin-2
82	TGFBI_OID01291 Transforming growth factor-beta-induced protein ig-h3
83	TGFBR3_OID01279 Transforming growth factor beta receptor type 3
84	THBS4_OID01268 Thrombospondin-4
85	TIE1_OID01270 Tyrosine-protein kinase receptor Tie-1
86	TIMD4_OID01298 T-cell immunoglobulin and mucin domain-containing protein 4
87	TIMP1_OID01224 Metalloproteinase inhibitor 1
88	TNC_OID01265 Tenascin
89	TNXB_OID01260 Tenascin-X
90	UMOD_OID01237 Uromodulin
91	VASN_OID01295 Vasorin
92	VCAM1_OID01257 Vascular cell adhesion protein 1
