# Supplementary material for: The FCGR2A Is Associated with the Presence of Atherosclerotic Plaques in the Carotid Arteries—A Case-Control Study
Source: J Clin Med. 2023 Oct 12;12(20):6480. doi: 10.3390/jcm12206480 (PMC10607679; doi:10.3390/jcm12206480)
Supplement: Supplementary file 1 [file jcm-12-06480-s001.zip › Supplementary Table S2.pdf]

Supplementary Table S2

Comparison of biomarker levels between patients with and without APCA (atherosclerotic plaques in the carotid arteries). Univariate logistic regression for association between APCA (dependent variable) and biomarkers (independent variable).

Nr	Biomarker	Patients with APCA N=320	Patients without APCA N=373	P Mann-Whitney	OR [95% CI]	P #
1	ANG	6.92 (6.63-7.25)	6.92 (6.64-7.23)	0.83	NA	NA
2	ANGPTL3	6.24 (5.96-6.56)	6.05 (5.8-6.29)	<0.001	3.09 [2.09-4.56]	<0.001
3	AOC3	4.16 (3.97-4.42)	4.04 (3.82-4.22)	<0.001	4.73 [2.84-7.89]	<0.001
4	APOM	6.88 (6.65-7.16)	6.92 (6.7-7.13)	0.27	NA	NA
5	C1QTNF1	4.93 (4.73-5.2)	4.86 (4.66-5.09)	0.003	1.68 [1.12-2.53]	0.012
6	C2	7.17 (6.96-7.34)	7.07 (6.82-7.28)	<0.001	1.81 [1.23-2.67]	0.003
7	CA1	5.74 (5.43-6.14)	5.64 (5.26-6.06)	0.61	NA	NA
8	CA3	1.82 (1.62-2.04)	1.61 (1.42-1.8)	<0.001	5.24 [3.28-8.4]	<0.001
9	CA4	2.36 (2.21-2.52)	2.37 (2.22-2.5)	0.49	NA	NA
10	CCL14	7.31 (7.06-7.5)	7.27 (7.06-7.5)	0.33	NA	NA
11	CCL18	6.76 (6.4-7.17)	6.47 (6.05-6.96)	<0.001	1.65 [1.33-2.04]	<0.001
12	CCL5	6.26 (5.81-6.66)	6.36 (5.95-6.78)	0.03	0.8 [0,64-0.98]	0.035
13	CD46	4.02 (3.87-4.2)	3.95 (3.79-4.1)	<0.001	2.78 [1.52-5.01]	0.001
14	CD59	1.05 (0.88-1.21)	0.93 (0.78-1.11)	<0.001	4.47 [2.49-8.03]	<0.001
15	CDH1	4.05 (3.83-4.29)	3.96 (3.75-4.16)	<0.001	1.76 [1.16-2.66]	0.007
16	CES1	3.71 (3.3-4.18)	3.44 (3.08-3.92)	<0.001	1.53 [1.25-1.9]	<0.001
17	CFHR5	8.26 (7.99-8.49)	8.21 (7.9-8.45)	0.045	1.34 [0.95-1.9]	0.092
18	CHL1	4.21 (4.05-4.38)	4.15 (3.96-4.35)	0.01	1.71 [1.04-2.8]	0.033
19	CNDP1	5.98 (5.72-6.23)	6.06 (5.79-6.29)	0.02	0.65 [0.47-0.89]	0.008

20	COL18A1	3.98 (3.8-4.19)	3.88 (3.73-4.08)	<0.001	2.5 [1.56-4.0]	<0.001
21	COMP **	8.46 ± 0.44	8.2 ± 0.43	<0.001	3.7 [2.56-5.37]	<0.001
22	CR2	7.76 (7.41-8.1)	8.01 (7.75-8.41)	<0.001	0.28 [0.2-0.39]	<0.001
23	CRTAC1	4.08 (3.74-4.44)	3.7 (3.42-4.0)	<0.001	4.11 [2.94-5.75]	<0.001
24	CST3	6.9 (6.68-7.19)	6.68 (6.42-6.97)	<0.001	4.4 [2.89-6.7]	<0.001
25	DEFA1	0.79 (0.58-0.93)	0.71 (0.56-0.9)	0.049	2.0 [1.34-2.99]	0.001
26	DPP4**	5.57 ± 0.35	5.53 ± 0.34	0.3	NA	NA
27	EFEMP1	5.75 (5.38-6.0)	5.46 (5.26-5.69)	<0.001	5.3 [3.4-8.19]	<0.001
28	ENG**	2.69 ± 0.22	2.68 ± 0.22	0.68	NA	NA
29	F11**	7.19 ± 0.31	7.14 ± 0.32	0.036	1.65 [1.03-2.64]	0.037
30	F7	4.59 (4.38-4.81)	4.55 (4.31-4.76)	0.05	NA	NA
31	FAP *	NA	NA	NA	NA	NA
32	FCGR2A	4.04 (3.79-4.31)	3.91 (3.58-4.14)	<0.001	1.94 [1.41-2.68]	<0.005
33	FCGR3B	4.63 (4.32-4.95)	4.65 (4.29-5.0)	0.57	NA	NA
34	FCN2	6.73 (6.38-7.02)	6.74 (6.36-7.09)	0.65	NA	NA
35	FETUB	2.8 (2.52-3.01)	2.84 (2.59-3.1)	0.01	0.62 [0.44-0.686]	0.004
36	GAS6**	5.5 ± 0.32	5.45 ± 0.31	0.09	NA	NA
37	GNLY*	NA	NA	NA	NA	NA
38	GP1BA	6.08 (5.84-6.27)	6.14 (5.93-6.34)	<0.001	0.45 [0.28-0.72]	0.001
39	ICAM1**	7.1 ± 0.35	6.96 ± 0.34	<0.001	3.14 [2.0-4.93]	<0.001
40	ICAM3	3.7 (3.53-3.88)	3.65 (3.46-3.82)	0.007	2.08 [1.23-3.51]	0.006
41	IGFBP3	4.68 (4.44-4.93)	4.79 (4.61-4.99)	<0.001	0.33 [0.21-0.5]	<0.001
42	IGFBP6	6.23 (6.05-6.52)	6.15 (5.96-6.38)	<0.001	2.68 [1.7-4.23]	<0.001
43	IGLC2	6.59 (6.3-6.95)	6.47 (6.16-6.75)	<0.001	1.82 [1.33-2.49]	<0.001
44	IL7R**	2.46 ± 0.46	2.51 ± 0.42	0.07	NA	NA
45	ITGAM*	NA	NA	NA	NA	NA
46	KIT**	4.86 ± 0.33	5.05 ± 0.32	<0.001	0.16 [0.1-0.27]	<0.001
47	LCN2	1.22 (0.99-1.45)	1.21 (0.97-1.5)	0.84	NA	NA

48	LILRB1**	3.06 ± 0.31	2.98 ± 0.28	<0.001	2.67 [1.58-4.51]	<0.001
49	LILRB2	4.09 (3.86-4.31)	4.0 (3.76-4.22)	0.001	1.89 [1.24-2.87]	0.003
50	LILRB5	5.34 (4.85-5.66)	5.28 (4.72-5.64)	0.36	NA	NA
51	LTBP2*	NA	NA	NA	NA	NA
52	LYVE1**	6.6 ± 0.35	6.59 ± 0.34	0.58	NA	NA
53	MBL2	9.73 (8.86-10.38)	9.65 (8.76-10.47)	0.75	NA	NA
54	MEGF9	4.47 (4.3-4.65)	4.48 (4.27-4.69)	0.48	NA	NA
55	MET**	2.94 ± 0.21	2.92 ± 0.23	0.12	NA	NA
56	MFAP5	2.16 (1.98-2.32)	2.06 (1.89-2.24)	<0.001	3.68 [2.06-6.55]	<0.001
57	NCAM1	4.5 (4.3-4.69)	4.43 (4.25-4.68)	0.05	NA	NA
58	NID1	5.17 (4.94-5.37)	5.17 (4.97-5.39)	0.68	NA	NA
59	NOTCH1**	3.99 ± 0.22	3.99 ± 0.22	0.68	NA	NA
60	NRP1**	1.54 ± 0.26	1.48 ± 0.24	0.002	2.56 [1.39-4.7]	0.003
61	OSMR	1.03 (0.89-1.18)	0.95 (0.79-1.09)	<0.001	7.52 [3.67-15.4]	<0.001
62	PAM**	2.59 ± 0.32	2.54 ± 0.29	0.04	1.69 [1.03-2.77]	0.036
63	PCOLCE	6.46 (6.12-6.83)	6.22 (5.99-6.47)	<0.001	3.07 [2.18-4.32]	<0.001
64	PLA2G7**	2.46 ± 0.32	2.38 ± 0.31	<0.001	2.26 [1.4-3.65]	0.001
65	PLTP**	2.64± 0.23	2.62 ±0.23	0.27	NA	NA
66	PLXNB2	2.22 (2.09-2.37)	2.15 (2.0-2.29)	<0.001	8.0 [3.88-16.48]	<0.001
67	PRCP	1.66 (1.5-1.85)	1.53 (1.33-1.72)	<0.001	3.62 [2.16-6.05]	<0.001
68	PROC	5.3 (5.03-5.51)	5.22 (4.96-5.44)	0.003	1.77 [1.2-2.61]	0.003
69	PRSS2	2.97 (2.74-3.32)	2.86 (2.62-3.17)	<0.001	1.98 [1.43-2.76]	<0.001
70	PTPRS	1.62 (1.46-1.73)	1.66 (1.49-1.8)	0.003	0.35 [0.18-0.7]	0.003
71	QPCT**	0.97 ± 0.29	1.02 ± 0.26	0.04	0.56 [0.32-0.98]	0.041
72	REG1A	7.2 (6.88-7.57)	7.0 (6.7-7.34)	<0.001	2.24 [1.66-3.02]	<0.001
73	REG3A*	NA	NA	NA	NA	NA
74	SAA4	4.97 (4.61-5.29)	4.81 (4.5-5.12)	<0.001	1.66 [1.26-2.2]	<0.001

75	SELL	9.02 (8.77-9.27)	9.2 (8.99-9.42)	<0.001	0.23 [0.14-0.36]	<0.001
76	SERPINA5**	9.44 ± 0.35	9.44 ± 0.35	0.86	NA	NA
77	SERPINA7**	5.73 ± 0.33	5.69 ± 0.35	0.1	NA	NA
78	SOD1*	NA	NA	NA	NA	NA
79	SPARCL1	3.68 (3.46-3.88)	3.6 (3.4-3.79)	0.001	2.5 [1.47-4.25]	0.001
80	ST6GAL1	4.32 (4.11-4.54)	4.23 (4.05-4.5)	0.007	1.44 [0.99-2.1]	0.055
81	TCN2**	5.21 ± 0.34	5.13 ± 0.31	0.001	2.11 [1.32-3.37]	0.002
82	TGFBI	9.28 (9.03-9.53)	9.25 (8.99-9.51)	0.62	NA	NA
83	TGFBR3	4.16 (3.93-4.38)	4.13 (3.92-4.34)	0.24	NA	NA
84	THBS4	5.44 (5.01-5.91)	5.19 (4.82-5.6)	<0.001	1.57 [1.26-1.95]	<0.001
85	TIE1**	2.37 ± 0.26	2.35 ± 0.26	0.31	NA	NA
86	TIMD4**	5.05 ± 0.45	4.97 ± 0.43	0.01	1.57 [1.11-2.21]	0.01
87	TIMP1	5.76 (5.59-5.9)	5.67 (5.52-5.84)	<0.001	2.62 [1.52-4.5]	<0.001
88	TNC	4.32 (3.96-4.69)	4.26 (3.99-4.65)	0.67	NA	NA
89	TNXB**	2.08 ± 0.23	2.14 ± 0.23	<0.001	0.33 (0.17-0.64)	0.001
90	UMOD*	NA	NA	NA	NA	NA
91	VASN**	2.37 ± 0.24	2.36 ± 0.24	0.47	NA	NA
92	VCAM1	5.18 (4.98-5.4)	5.12 (4.94-5.29)	0.005	2.19 [1.34-3.56]	0.002

Columns 3-5 contain comparison of biomarker levels between patients with and without APCA (atherosclerotic plaques in carotid arteries). Columns 6-7 contain results of univariate logistic regression for association between APCA (dependent variable) and biomarkers (independent variable)

Results are presented in NPX units (*Normalized Protein eXpression*)

Data presented as median with interquartile range ((IQR) 1st quartile–3rd quartile). Differences between the groups were tested with the Mann–Whitney test.

* Data not analyzed- >25% of samples below LOD (limit of detection)

** normal distribution. data was presented as mean ± standard deviation. Differences between the groups were tested with T-Student test.

p value for association between biomarker and APCA in univariate logistic regression

APCA- atherosclerotic plaques in carotid arteries
